# Nitrite oxidizing bacteria (NOB) dominating in nitrifying community in full-scale biological nutrient removal wastewater treatment plants

**DOI:** 10.1186/s13568-017-0328-y

**Published:** 2017-01-23

**Authors:** Qian Yao, Dang-Cong Peng

**Affiliations:** 1 School of Environmental and Municipal Engineering, Xi’an University of Architecture and Technology, Yanta Road 13, Xi’an, 710055 China; 2 Key Laboratory of Northwest Water Resource Environment and Ecology, Ministry of Education, Xi’an, 710055 China

**Keywords:** Nitrifying community, Biological nutrient removal, FISH, Activity, Dominating

## Abstract

Nitrification activities and microbial populations of ammonium oxidizing bacteria (AOB) and nitrite oxidizing bacteria (NOB) were investigated in 10 full-scale biological nutrient removal wastewater treatment plants in Xi’an, China. Aerobic batch tests were used to determine the nitrifying activities while fluorescence in situ hybridization was used to quantify the fractions of AOB and NOB in the activated sludge. The results showed that nitrifying bacteria accounted for 1–10% of the total population. *Nitrosomonas* and *Nitrospira* were the dominant bacteria for AOB and NOB respectively. Moreover, the average percentage of AOB was 1.27% and that of NOB was 4.02%. The numerical ratios of NOB/AOB varied between 1.72 and 5.87. The average ammonium uptake rate and nitrite uptake rate were 3.25 ± 0.52 mg (NH_4_^+^–N)/g(VSS) h and 4.49 ± 0.49 mg (NO_2_^−^–N)/g(VSS) h, respectively. Correspondingly, the activity of NOB was 1.08–2.00 times higher than that of AOB. Thus, NOB was the dominating bacteria in nitrifying communities. The year-round data of Dianzicun (W6) also expressed a similar trend. Since NOB had higher activities than that of AOB, a large nitrite oxidation pool could be formed, which guaranteed that no nitrite would be accumulated. Therefore, stable nitrification could be achieved. A conceptual model was proposed to describe the population variation of AOB and NOB in a nitrifying community.

**Electronic supplementary material:**

The online version of this article (doi:10.1186/s13568-017-0328-y) contains supplementary material, which is available to authorized users.

## Introduction

Nitrification is of great importance for nitrogen removal from municipal wastewater in the biological nutrient removal process (BNR) employed in waste water treatment plants (WWTPs). In nitrification, ammonium is firstly oxidized to nitrite via ammonia-oxidizing bacteria (AOB), and then to nitrate by nitrite-oxidizing bacteria (NOB). However, due to low biomass yield and sensitivity to environmental factors, nitrifying bacteria only account for a small fraction of total biomass. Although nitrifying bacteria populations are generally within the range of 4–6% of biomass for adequate nitrification in nutrient removal facilities (Nielsen et al. 2004), a wide variation of the fraction of nitrifying bacteria in microbial communities has been reported. It varies from 0.39% in activated sludge (Dionisi et al. 2002), to 9% in a nitrifying activated sludge SBR reactor (Li et al. 2007), and even to over 18% in a combined activated sludge and rotating biological contactor (You et al. 2003). The difference of the percentage of nitrifying bacteria may be affected by operational conditions and influent qualities.

Theoretically, the numerical ratio of AOB to NOB in a balanced nitrifying system should be 2:1 according to thermodynamics and electron transfer (Arciero et al. 1991; Hooper et al. 1997; Mari et al. 2012), which means that AOB should be the dominant bacteria in a nitrifying community. Li et al. (2007) found that the AOB to NOB ratio in a sufficient nitrification process was 2.2–2.7. In a similar result, You et al. (2003) reported the percentage of AOB was 2.0–3.5 times higher than NOB. However, there were some exceptions demonstrating that, from time to time, a disproportion in the ratios of AOB/NOB existed. Ramdhani et al. (2013) investigated the nitrifying bacteria communities at two full-scale domestic wastewater treatment plants in South Africa: lower AOB/NOB ratios were detected, 1.0–1.5 in Kingsburgh WWTP and 0.8–1 in Darville WWTP. Harms et al. (2003) found NOB (*Nitrospira*) could reach more than three times higher than AOB in a municipal wastewater treatment plant. Moreover, in the lab and pilot studies of Mari et al. (2012), an elevated NOB/AOB ratio (3–4) was observed in an aerobic granular sludge sample. These controversial data suggest that more investigations are needed.

Due to the sequential oxidation property, the growth balance between AOB and NOB plays a key role in optimization of a nitrifying community. If AOB grows more quickly than NOB, and the ammonium oxidizing rate is higher than nitrite oxidizing rate, nitrite as an intermediate will be easily accumulated. Nitrite is toxic to aquatic ecosystems and poses potential threats to human health security. Furthermore, nitrite will be converted under anoxic condition by *Nitrosomonas* to nitrous oxide (N_2_O) (Colliver and Stephenson 2000), which is a lethal greenhouse gas (GHG) causing ozone depletion. Therefore, fully understanding the population and interaction of AOB and NOB in the nitrifying community is very important to optimize nitrification in biological nutrient removal plants.

In this study, 10 full-scale biological nutrient removal plants in Xi’an, China, were investigated in terms of process efficiency, nitrification activity and the nitrifying community. Nitrification activity in each WWTP was evaluated by aerobic batch tests using fresh activated sludge. The fractions of AOB and NOB and the dominating bacteria were determined by fluorescence in situ hybridization (FISH). The objectives were to attempt to answer the following questions: ➀ How do AOB and NOB distribute in full-scale biological nutrient removal WWTPs? ➁ What is the real ratio of AOB and NOB in nitrifying communities in treatment plants? ➂ How do nitrifying bacteria communities interact with operational processes and parameters?

## Materials and methods

### Plants and sampling

Ten biological nutrient removal plants in Xi’an, China were investigated: Dengjiacun (W1), Dengcun (W2), Beishiqiao (W3), Bshiq (W4), Fangzhicheng (W5), Dianzicun (W6), Yuanlecun (W7), Liucunbao (W8), Gaoxin (W9) and Chang’an (W10). They were located in different areas and received both domestic and industrial wastewaters. W1 and W2 shared one sewer and had the same influent composition. Information related to plant process configurations, influent/effluent wastewater compositions and operation parameters was directly obtained by reviewing plant documents, interviewing plant operators and visiting WWTPs facilities. Operational data and treatment efficiencies are compiled in Table 1.

**Table 1:** Summary of the overall operating dada of the waste water treatment plants

Plant	Process	Operational parameters									Influent			Effluent		
		Flow rate (10^4^m^3^/d)	Tem (°C)	SRT (d)	HRT (h)	SS (mg/L)	VSS (mg/L)	SV_30_	Sludge recycle ratio	pH in aeration basin	COD (mg/L)	TN (mg/L)	NH_4_^+^-N (mg/L)	COD (mg/L)	TN (mg/L)	NH_4_^+^-N (mg/L)
W1	A^2^O	14.5	23	10	11–12	3600	2500	26	90	7.5	500–600	58	45	30	13	≤1
W2	MAO	14.5	23	10	11–12	4000	2800	50	100	7.5	500–600	58	45	30	13	3
W3	Oxidation ditch	15	23.2	16	12	6000	4080	88	95	7.2	368	43.29	35.14	21	4.36	0.68
W4	MAO	15	25	15	12	5800	3900	90	95	7.2	208	46.75	39.12	23.67	16.95	11.56
W5	Oxidation ditch	15	24.2	17–19	21	5700	3000	88	100	7.7	301	39	27.57	22	7.06	0.363
W6	A^2^O	25	23.1	23	12.5	4000	2500	70	100	7.6	620	47.88	30.71	18	12.66	0.18
W7	A^2^O	20	24.17	19	15	7000	4500	88	80–85	7.6	442.71	43.7	32.27	17.9	7.68	≤1
W8	A^2^O	10	25	10	16	5800	3000	87	80	7.5	700	48	25–30	22	8	0.1
W9	Oxidation ditch	6.8	20	17	18	4800	2400	50	80	7.5	550	48	40	22	10	0.5–1
W10	Oxidation ditch	10	20	15	20	4500	2500	65	60	7.5	550	65	50	20	8	1

Fresh activated sludge was collected from the final stage of the aeration basin in each plant on September 5th, 15th and 25th, 2015, respectively. During the survey period, water temperature varied from 20 to 25 °C. Samples in W6 were collected monthly for over a year (from January 2015 to March 2016). All samples were stored in an ice box and kept at 4 °C during transportation. For fluorescence in situ hybridization analysis, sludge was fixed immediately upon arrival in 4% paraformaldehyde (PFA) for 3 h at 4 °C and stored in phosphate-buffered saline/96% Ethanol (1:1, vol/vol) at −20 °C. Additional sludge aliquots were tested immediately in the lab for nitrifying activity.

### The activity of nitrifying bacteria

Monitoring the activity of nitrifying bacteria was carried out by two methods: oxygen uptake rates (OUR) and substrate uptake rates (SUR).

Oxygen uptake rates were measured using a conventional respirometer (Strathkelvin 782) with 0.5 mL Mitocell (MT200A). Allylthiourea (ATU, 5 mg/L) was added to inhibit the ammonia to be oxidized to nitrite. Sodium chlorate (NaClO_3_, 2 g/L) was added to inhibit the oxidation of nitrite to nitrate. The temperature was maintained at 25 °C for all respiratory analysis. Prior to analysis, samples were elutriated and aerated to remove all soluble substances.

For the SURs test, ammonium chloride (20 mg NH_3_–N/L) and sodium nitrous acid (30 mg NO_2_–N/L) were used as the substrate to measure ammonium uptake rates (AUR) and nitrite uptake rates (NUR), respectively. During the whole test, temperature was maintained as the same for the OUR test, 25 °C. NaHCO_3_ was added to ensure a stable pH. The mixed liquor was purged with air and spiked with substrate in the presence of the inhibitor. Then, samples were taken every 10 min and NH_3_–N and NO_2_–N were measured to calculate AUR and NUR respectively.

### Chemical analysis

Ammonium, nitrite, nitrate, mixed liquor suspended solids (MLSS) and volatile MLSS (MLVSS) were determined according to Standard Methods (APHA 1995). Dissolved oxygen (DO) concentration was measured with an oxygen dissolving meter (SG6-FK10, Mettler Toledo).

### Florescence in situ hybridization (FISH)

Table 2 summarizes the 16S rRNA-targeted oligonucleotide probes used in this study. Probe EUBmix (an equimolar mixture of EUB338, EUB338IIand EUB338III), labeled with Cy5, was used to target almost all bacteria. Probe AOBmix (a mixture of Nso1225, NEU, NmV and Cluster6a192), labeled with Flous, was used to target the AOB. Probe NOBmix (a mixture of Ntspa662, NIT3 and Ntspa712), labeled with Cy3, was used to detect NOB. Moreover, Nsm156 was specific for *Nitrosomonas* spp.; Nsv443 was specific for *Nitrosospira* spp.; Ntspa662 was specific for *Nitrospira* spp. and NIT3 was specific for *Nitrobacter* spp.

**Table 2:** rRNA-targeted oligonucleotide probe used in this study

Probe	Specific	Sequence (5′–3′)	FA^a^ (%)	Reference
EUB		GCTGCCTCCCGTAGGAGT		
EUBII	*Almost all bacteria*	GCAGCCACCCGTAGGTGT	0–80	Daims et al. (1999)
EUBIII		GCTGCCACCCGTAGGTGT		
Nso1225	*Betaproteobacterial ammonia*-*oxidizing bacteria*	CGCCATTGTATTACGTGTGA	35	Mobarry et al. (1996)
NEU	*Most halophilic and halotolerant Nitrosomonas* spp.	CCCCTCTGCTGCACTCTA	40^b^	Wagner et al. (1995)
NmV	*Nitrosococcus mobilis*	TCCTCAGAGACTACGCGG	35	Pommerening et al. (1996)
Cluster6a192	*Nitrosomonas oligotropha lineage*	CTTTCGATCCCCTACTTTCC	35	Adamczyk et al. (2003)
Nsm156	*Nitrosomonas* spp., *Nitrosococcus mobilis*	TATTAGCACATCTTTCGAT	5	Mobarry et al. (1996)
Nsv443	*Nitrosospira* spp.	CCGTGACCGTTTCGTTCCG	30	Mobarry et al. (1996)
Ntspa712	*Phylum Nitrospirae*	CGCCTTCGCCACCGGCCTTCC	50^c^	Daims et al. (2001)
Ntspa662	*Genus Nitrospira*	GGAATTCCGCGCTCCTCT	35	Daims et al. (2001)
NIT3	*Genus Nitrobacter*	CCTGTGCTCCATGCTCCG	40	Wagner et al. (1996)

^a^ *FA* formamide

^b^ NEU can also be used with 35% FA

^c^ Ntspa712 can also be used with 35% FA, especially if combined with Ntspa662

The hybridization of the activated sludge sample was performed according to the standard hybridization protocol described by Amann et al. (1990). 8 µL of the hybridization buffer (0.9 M NaCl, 20 mM Tris–HCl, 0.01% SDS and formamide [concentration has been given in Table 2]) and 1 µL fluorescent probe (50 ng/µL) were placed on each well and mixed gently with a tip. The slide was then transferred into the prepared equilibrated chamber and hybridized for 1.5–2 h at 46 °C, after which it was washed at the hybridization temperature for 10 min in a washing buffer (20 mM Tris–HCl, 5 mM EDTA and NaCl) and rinsed with distilled water for 5 s. After final air drying, the slide was mounted with a drop of Citiflour (Sigam, USA), covered with a coverslip and viewed immediately.

### Microscope and image analyze

A confocal laser scanning microscope (CLSM; Leica SP8, Germany) equipped with one Ar-ion laser (488 nm, for detection of Flous) and two HeNe lasers (552 and 638 nm, for detection of Cy3 and Cy5 respectively) was used to examine the microbial community. Both stages of image combining and processing were performed with the process tools of the software delivered by the CLSM supplier. The image analysis was determined by using the software package PaintShopPro (Jasc, Eden Prairie, MN, USA).

For community testing, total 60 views were obtained for each WWTP (ten views for each well, two wells for each sample and three samples for each WWTP). All images were first processed with blur (or out-of-focus) removing, then three colors (Cy3-Red; Cy5-Blue; Flous-Green) were counted separately for each image. The proportions and numbers of the targeted nitrifiers were calculated according to Li et al. (2007) and Manser et al. (2006).

## Results

### Operational data and performance

Operating parameters and average influent and effluent characteristics for all WWTPs are shown in Table 1. Significant variations in influent characteristics (COD, TN and NH_3_–N) were recorded. COD in W6 reached as high as 620 mg/L, while in W4, it was as low as 260 mg/L during the survey. TN in the influent varied between 39 and 65 mg/L and NH_3_–N between 25 and 50 mg/L. The fluctuations of influent characteristics reflected the developing sewer systems in the region.

Due to higher COD/N ratios in the influent, high TN removal rates (63.74–89.93%) were achieved in all of the plants investigated. Research has shown that simultaneous nitrification and denitrification (SND) will be stimulated in an Oxidation Ditch, which benefits high TN removal (Zhou et al. 2013). In this research, similar results were observed. TN removal rates in an Oxidation Ditch seemed to be higher than that in A^2^/O. In addition, although a wide range of SRT (10–23 days) and HRT (11–20 h) were employed, NH_3_–N concentrations in the effluent were less than 3 mg/L in all plants except W4, in which DO concentration in the aeration basin was low. These results suggest that good nitrification was achieved. Excellent nitrification performance meant that stable nitrifying communities were built and a solid foundation for the investigation was established.

### Morphology and spatial distribution of nitrifying bacteria

**Fig. 1:**
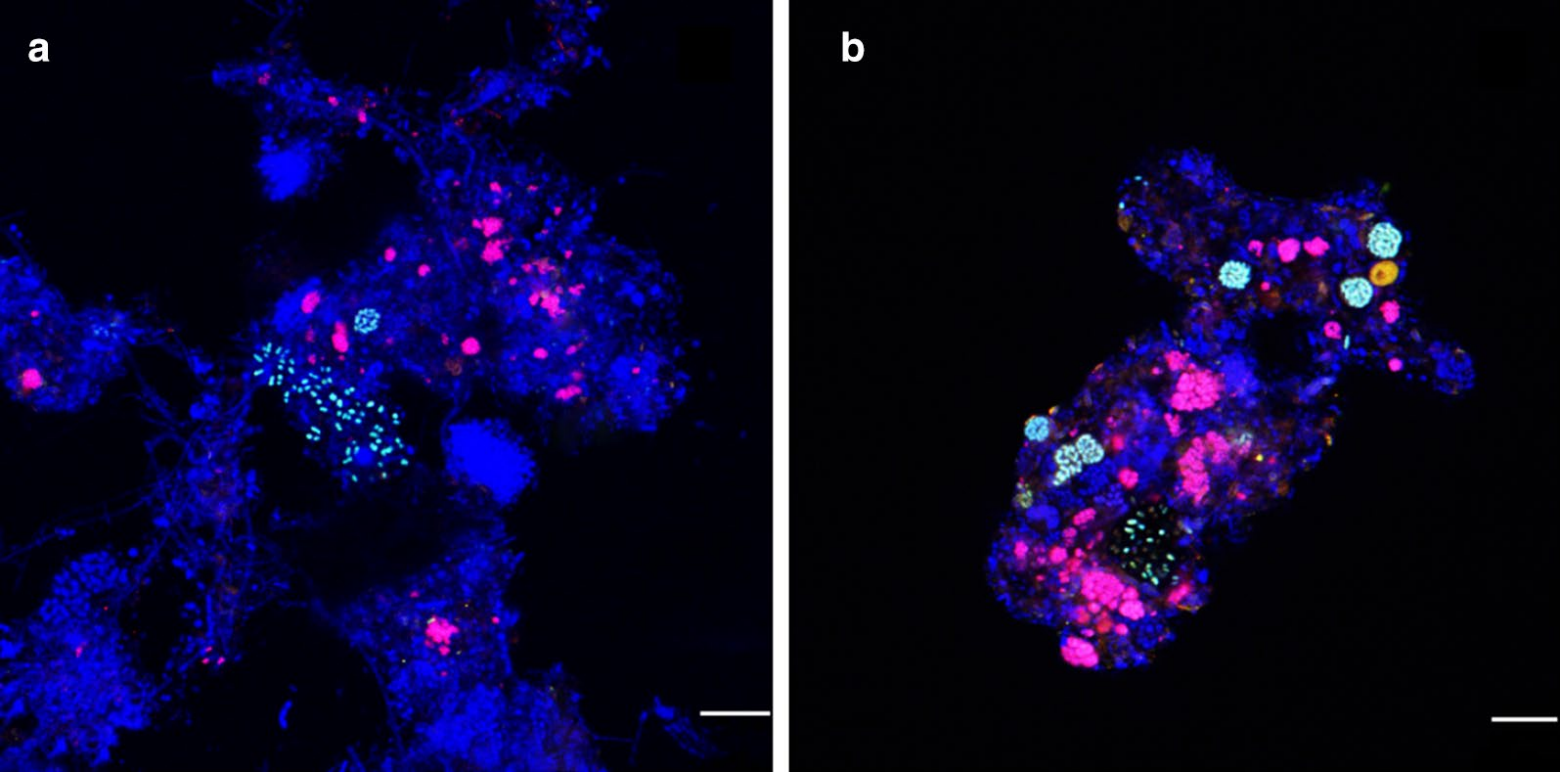
In situ hybridization of activated sludge. In situ hybridization of activated sludge with Cy5-labeled probe EUBmix, Flous-labeled probe AOBmix and Cy3-labeled probe NOBmix (**a** is the activated sludge from W7 and **b** is from W6). *Blue* EUBmix-stained Eubacteria; *cyan* AOBmix-stained AOB; *carmine* NOBmix-stained NOB; *Bar* 10 μm

To investigate the spatial distribution and morphology of nitrifying bacteria, FISH was carried out for all sludge samples. The results showed that nitrifying bacteria were not uniformly distributed in the activated sludge (Fig. 1 for W7 and W6. For others, see Additional file 1: Figure S1). Both AOB and NOB were found to be aggregated in spherical or irregular microcolonies. NOB exhibited in a denser and smaller microcolony (2–12 μm), but AOB exhibited in a looser and bigger microcolony (5–25 μm).

Concerning space distribution, nitrifying bacteria grew inside the flocs while heterotrophic bacteria grew outside. This spatial distribution character reflected their competition for oxygen in the bulk. Typically, heterotrophic bacteria have a higher oxygen affinity and growth rate than that of nitrifying bacteria (Pambrun et al. 2006). Therefore, heterotrophic bacteria will grow quickly and are present outside the flocs. In addition, NOB colonies were located in the vicinity of AOB colonies according to the results. This co-occurrence of AOB and NOB has been confirmed in the studies of nitrifying bacteria in biofilm and activated sludge (Okabe et al. 1996), which can minimize the diffusion distance of the intermediate and reflect the syntrophic association between AOB and NOB (Mobarry et al. 1996).

### Activity and quantity of nitrifying bacteria in ten WWTPs

**Fig. 2:**
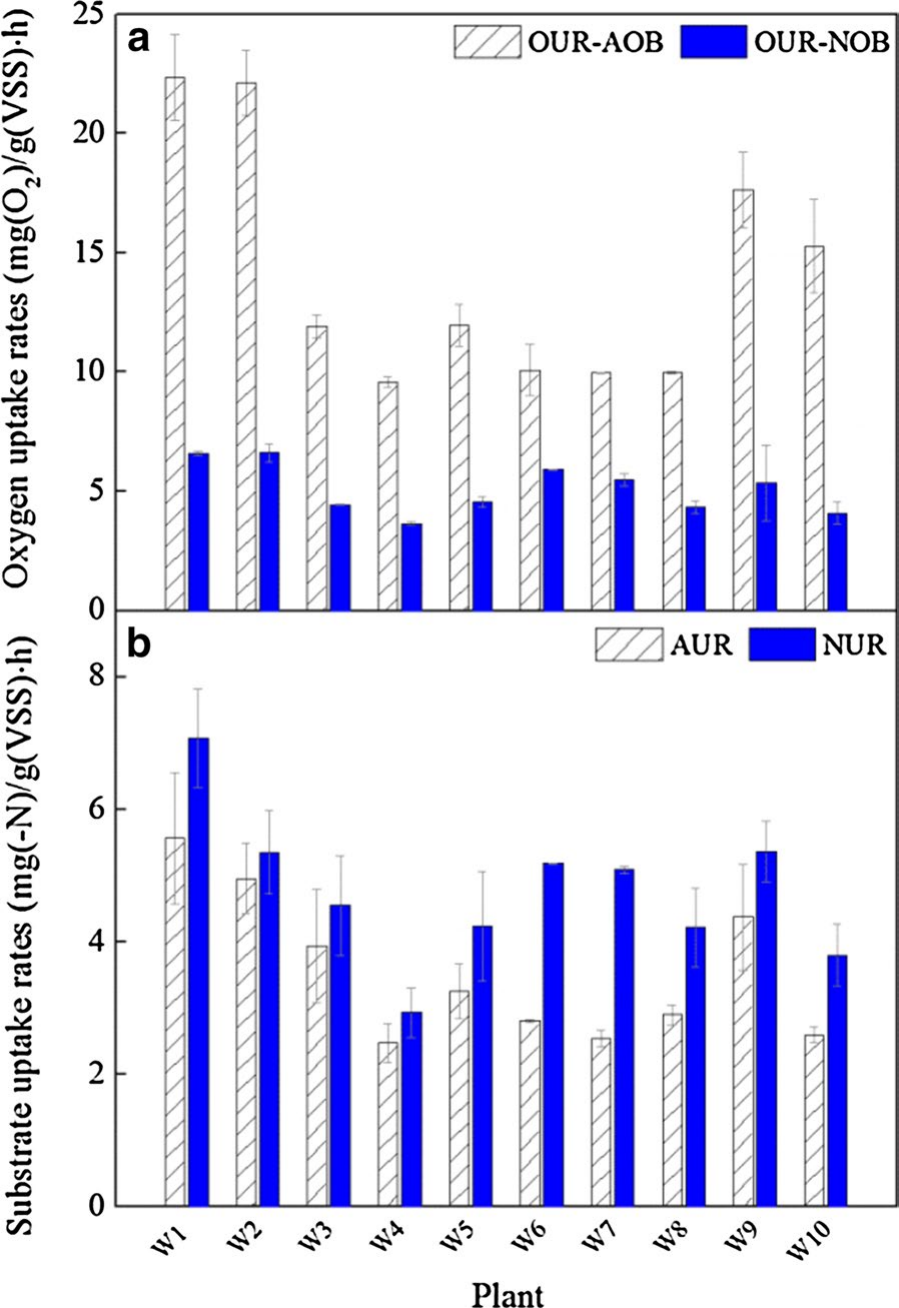
Nitrifying activity in 10 WWTPs. **a** oxygen uptake rates of AOB (OUR-AOB) and NOB (OUR-NOB); **b** ammonium uptake rates for AOB (AUR) and nitrite uptake rates for NOB (NUR)

Nitrification activities in 10 WWTPs are shown in Fig. 2. The OURs of AOB ranged from 9.58 to 22.35 mg(O_2_)/g(VSS) h, and were 1.70–3.74 times higher than that of NOB (Fig. 2a). In the sequential reaction, the oxygen demand for the ammonium oxidation step and nitrite oxidation step are usually 3:1, thus AOB had a higher OUR than NOB. However, stoichiometrically, six electrons are needed for oxidizing one mole of ammonium to nitrite, but only two for nitrite to nitrate. This may result in that AUR was lower than NUR in turn. The direct detection of AUR and NUR also reflected the same trend (Fig. 2b). The average AUR and NUR were 3.25 ± 0.52 mg (NH_4_^+^–N)/g(VSS) h and 4.49 ± 0.49 mg(NO_2_^−^–N)/g(VSS) h, respectively. The highest AOB and NOB activities were observed in W1 while W4 had the lowest AUR and NUR, in which DO concentration in the aeration basin was measured to be 0.5-1 mg/L.

**Fig. 3:**
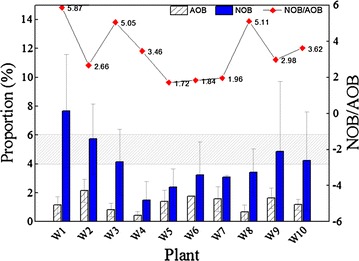
Nitrifying bacterial quantity and NOB/AOB ratio for 10 WWTPs

The relative amounts of the targeted bacteria were calculated as the percentage of the total biomass by using FISH. The results are shown in Fig. 3. Nitrifying bacteria accounted for 1–10% (average, 5.29 ± 2.11%) of the total biomass in the WWTPs surveyed. Five of them ranged from 4 to 6%, a typical nitrifying bacteria proportion in biological nutrient removal plants as Nielsen et al. (2004) reported. The nitrifying bacteria in W4 presented the lowest percentage (1.82 ± 1.69%) with 0.43 ± 0.28% of AOB and 1.49 ± 1.28% of NOB in biovolume. Whereas W1 had the highest percentage of nitrifying bacteria of 8.97 ± 3.86% and AOB and NOB accounted for 1.15 ± 0.57 and 7.67 ± 3.91% respectively. The quantities of nitrifiers were consistent with the nitrifying activities in batch tests. The higher percentage of a targeted microbe in a mixed culture, the higher activity of the mixed culture will be observed. However, in our results, no clear linear correlation was suggested. In addition, FISH results showed that the ratios of NOB/AOB varied from 1.72 to 5.87. NOB dominated in the nitrifying bacteria community in all the WWTPs surveyed.

### Activity and quantity of nitrifying bacteria in W6

**Fig. 4:**
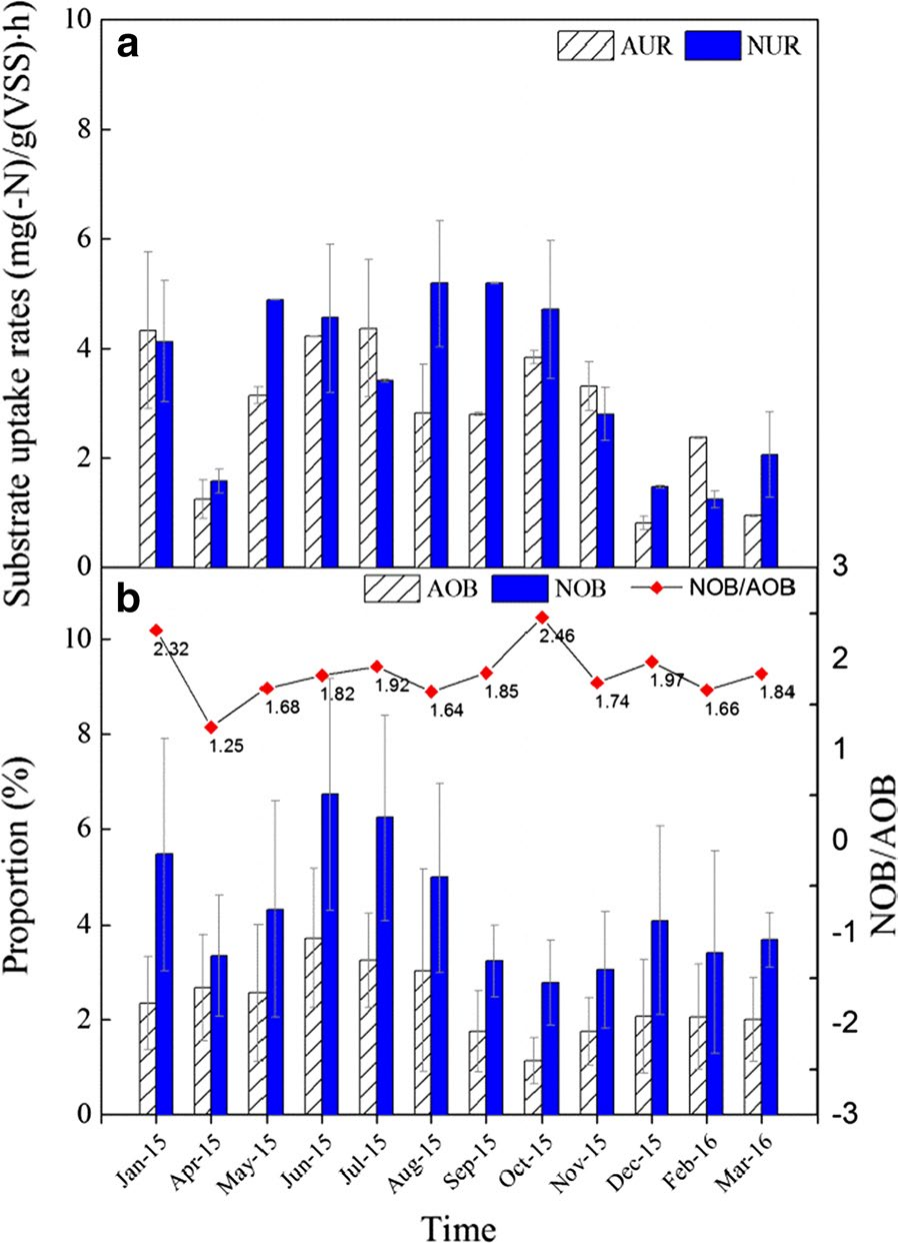
Year-round data for the activity and quantity of nitrifying bacteria in W6. **a** nitrifying activity; **b** proportion of AOB and NOB

FISH test displayed a significant amount of AOB (average of 2.36%) and NOB (average of 4.28%) from January 2015 to March 2016 (Fig. 4) in W6. The NOB/AOB ratio fluctuated throughout the study period and ranged from 1.25 to 2.46 (Fig. 4b). An overall higher NOB proportion was noted when compared with AOB. During the investigated periods, the higher activity of nitrifying bacteria occurred from June to August 2015. This may have been caused by the higher summer temperature (20–30 °C), which is sufficient to ensure the complete growth of nitrifying bacteria and the establishment of stable communities of AOB and NOB (Hao et al. 2002). As expected, both of the AUR and NUR decreased during winter (from December to February). Since NOB is more sensitive to lower temperature, the NUR decreased 48% when compared with that in summer, while AUR only decreased 34%. Moreover, the proportion of AOB and NOB also showed a remarkable decline in the winter. However, it must be addressed here that the proportion of NOB declined no more than AOB. This was caused by the specific growth rate of NOB which was higher than that of AOB, between 5 and 15 °C. It is known that NOB dominates AOB at temperatures below 15 °C (Zhu et al. 2008).

### Population structure of nitrifying bacteria

Different combinations of 16SrRNA-targeted oligonucleotide probes were used to characterize nitrifying population structures in the activated sludge for either *Nitrosomonas* spp. or *Nitrosospira* spp. of AOB and *Nitrobacter* spp. or *Nitrospria* spp. of NOB. The results are shown in Additional file 1: Figure S2. In most WWTPs, *Nitrosomona*s and *Nitrospria* were found to be the dominant bacteria with a fraction of 0.42–1.53 and 1–4.96% of total biomass, which accounted for 63–97.70% of AOB and 60–91.71% of NOB, respectively. *Nitrosospira*-like AOB and *Nitrobacter*-like NOB could only be detected in some samples and accounted for 0–0.35 and 0–0.53% of the total biomass (Additional file 1: Figure S3). Additionally, it is worth noting that neither *Nitrospira* nor *Nitrobacter* was the dominant nitrite oxidizer in W3, suggesting that some other species of NOB was also important in the activated sludge. In the studies of Lücker et al. (2014) and Saunders et al. (2015), *Nitrotoga* were numerically abundant in the activated sludge plants in Austria and Denmark, which challenged the previous assumptions that *Nitrospira* was the dominant nitrite-oxidizers in activated sludge.

## Discussion

### Dominant bacteria responding to different treatment processes

Nitrifying community structures in activated sludge of 10 WWTPs were analyzed by FISH. Four major groups of nitrifying bacteria which were frequently reported to exist in WWTPs were detected. According to the results, no significant difference was revealed in the dominant nitrifying bacteria in 10 WWTPs. *Nitrosomonas* spp. and *Nitrospira* spp. were found to be the dominant nitrifying bacteria in most systems. This finding supports the point that *Nitrospria*-like NOB, as a K strategist, could thrive in a low nitrite environment, especially in most municipal WWTPs (Dionisi et al. 2002; Freitag et al. 2005; Off et al. 2010). Additionally, consistent with most previous studies (Wagner et al. 1996), *Nitrosospira* was not the most important bacteria in WWTPs, and was not even detected in some samples. *Nitrosomonas* (such as *N. europaea)*, which grow more quickly than *Nitrosospira* spp. (Siripong and Rittmann 2007) can outcompete *Nitrosospira* as the prevailing group in the activated sludge of full-scale wastewater treatment plants.

In the study of Wang et al. (2014), nine operational and environmental variables were tested to determine the factors that shaped microbial community structure in WWTPs. Among them, DO, temperature and ammonium concentration were the important variables strongly influencing the microbial community and this result was consistent with the findings of Wells et al. (2009), Gregory et al. (2010) and Szukics et al. (2010). However, in the 10 WWTPs surveyed in this study, almost all WWTPs were operated similarly involved ammonium concentration, temperatures and DO concentrations (about 2 mg/L in the 10 WWTPs). Moreover, although they were operated with different treatment processes, they all use the activated sludge system. Therefore, there was no selection pressure which gave rise to the change of domination bacteria in the nitrifying community. But, it worth noting, there were still some exceptions in W3 and W5 where *Nitrospira* and *Nitrosomonas* were not the dominating bacteria respectively. This result may be caused by the fact that selection had already begun in the sewers, and there were some other organisms acting as a seed for selection.

### Activity of nitrifying bacteria

Activity is generally used to describe the maximum substrate utilization potential of targeted microorganisms in a community. The higher percentage of a targeted microbe in a mixed culture, the higher activity of the mixed culture will be observed.

With FISH technology, the number of a targeted microbe in a mixed culture can be counted. In our study, AOB cell numbers ranged between 4.3 × 10^9^ and 2.42 × 10^10^ cell/L and NOB was in the range of 1.49–7.67 × 10^10^ cell/L. Therefore, the real activity of the targeted microbe can be estimated as the following equations:

Specific activity for mass [mg (N)/g AOB (NOB) h] = $\frac{{\text{Activity}{\kern 1pt} \text{ (mg N/gVSS h)}}}{{\text{Fraction}{\kern 1pt} {\kern 1pt} {\kern 1pt} \text{of}{\kern 1pt} {\kern 1pt} \text{AOB}{\kern 1pt} {\kern 1pt} {\kern 1pt} \text{or}{\kern 1pt} {\kern 1pt} {\kern 1pt} \text{NOB}{\kern 1pt} {\kern 1pt} {\kern 1pt} {\kern 1pt} {\kern 1pt} {(\% biomass)}}}$.

Specific activity for a cell [mg (N)/cell h] = $\frac{{\text{Activity}{\kern 1pt} \text{ (mg N/gVSS}\;\text{h)}}}{{\text{Number}{\kern 1pt} {\kern 1pt} {\kern 1pt} \text{of}{\kern 1pt} {\kern 1pt} \text{AOB}{\kern 1pt} {\kern 1pt} {\kern 1pt} \text{or}{\kern 1pt} {\kern 1pt} {\kern 1pt} \text{NOB}{\kern 1pt} \text{ (cell/L)}{\kern 1pt} {\kern 1pt} {\kern 1pt} {\kern 1pt} }}$.

Table 3 shows the specific activities of nitrifying bacteria reported in the relevant literatures and our study. The specific activity for cells varied remarkably in the WWTPs. The average specific activity for cells of AOB in our study was 22.99 ± 11.01 fmol-N/cell h, which was consistent with the results reported by Daims et al. (2001) and Limpiyakorn et al. (2005). However, it was higher than that reported in lab-scale reactors. In fact, microorganisms have different growth rates during different growth phases. If longer SRT is employed, the bacteria as a whole will grow in a “stationary phase”. The lower activity will be observed. Therefore, due to longer SRTs (20–22 days), the activities of AOB in lab-scale WWTPs (Hanaki et al. 1990; Copp and Murphy 1995; Sun 2004) were lower than in our study. Similarly, in the surveyed WWTPs, W6 and W7 had longer SRTs (20 and 19 days) and lower specific activities (159.79 and 160.33 NH_4_^+^–N/gAOB h, respectively) were detected. Whereas both W1 and W8 had shorter SRTs (10 days), the specific activities of AOB were 483.14 and 434.62 NH_4_^+^–N/gAOB h, respectively. As for NOB, the average specific activity for cells was 9.43 ± 2.79 fmol–N/cell h, which was nearly the same as the value Fujita et al. (2010) reported.

**Table 3:** Specific activity of AOB and NOB in lab-scale and full-scale WWTPs

Reference	AOB		NOB	
	(mg-N/gAOB/h)	(fmol-N/cell/h)	(mg-N/gNOB/h)	(fmol-N/cell/h)
Our study (average)	321.94 ± 154.19	22.99 ± 11.01	132.028 ± 39.06	9.43 ± 2.79
Other studies				
*Full*-*scale*				
Limpiyakorn et al. (2005)	–	0–49.6	–	–
Harms et al. (2003)	–	7.7 ± 6.8	–	–
Daims et al. (2001)	–	16–43	–	–
Fujita et al. (2010)	–	1.1–11.9	–	2.4–21.6
Belser and Schmidt (1980)	–	9–123	–	–
*Lab*-*scale*				
Copp and Murphy (1995)	175	–	–	–
Sun (2004)	109	–	–	–
Hanaki et al. (1990)	70	–	–	–

### NOB dominating in nitrifying community

**Fig. 5:**
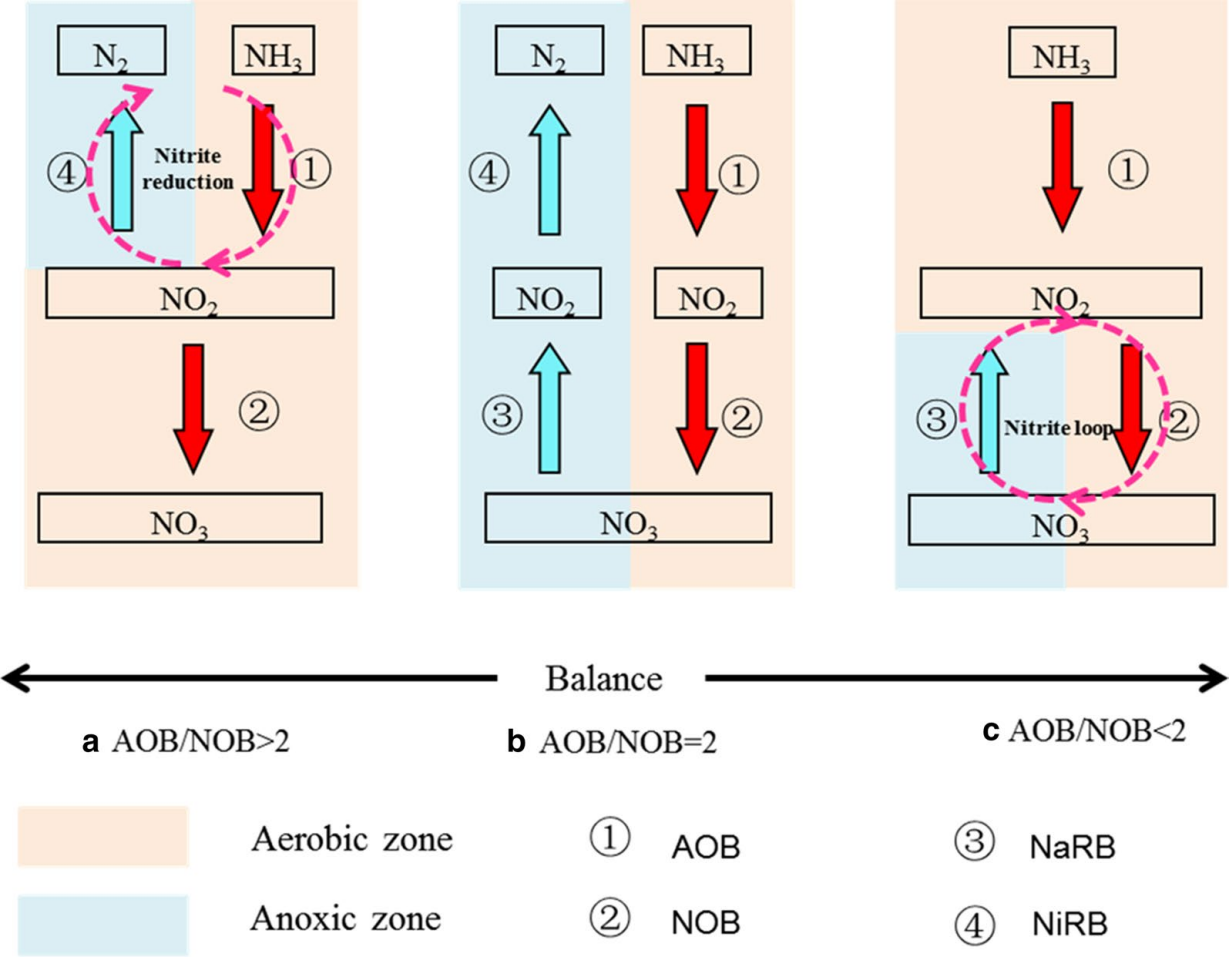
**a** is a situation that the numerical ratio of AOB/NOB is more than 2; **b** is a situation that the numerical ratio of AOB/NOB is 2; **c** is a situation that the numerical ratio of AOB/NOB is less than 2

Our research demonstrated an elevated NOB/AOB ratio (1.25–5.9) in 10 full-scale biological nutrient removal wastewater treatment plants, which was much higher than the expected ratio of 0.5 based on the electron transfer for bacterial growth in nitrification. Why was NOB abundant with so high a percentage in a nitrifying community? There must be an access that the growth of NOB does not rely on the nitrite provided by AOB. In order to explain this phenomenon, we constructed a conceptual model to describe the bacterial growth balance in a nitrifying community (Fig. 5). Nitrification is normally coupled with denitrification to convert ammonium to dinitrogen gas. If nitrification and denitrification take place independently, nitrite produced by AOB will be used completely by NOB. The growth balance of AOB and NOB can be reached. The numerical ratio of AOB/NOB should be 2 as shown in Fig. 5b. Where there is a competition between nitrite oxidation by NOB and nitrite reduction by nitrite reducing bacteria (NiRB), part of nitrite produced by AOB will be reduced to nitrogen gas by NiRB. The substrate (nitrite), which should be used for NOB, will be reduced. The numerical ratio of AOB/NOB should be more than 2 (Fig. 5a). In the studies of You et al. (2003), Fujita et al. (2010) and Mari et al. (2012), AOB/NOB ratios were revealed to be 3–10 in nitrifying communities at wastewater treatment plants. However, there is also a case where nitrite oxidation is coupled with nitrate reduction as described by Mari et al. (2012). A nitrite loop will be formed. Additional substrate (nitrite) could be provided by nitrate reducing bacteria (NaRB) for NOB growth. The number of NOB in the community will be more than that in Fig. 5b, and the ratio of AOB/NOB will be less than 2 as shown in Fig. 5c.

This model can well explain the higher percentage of NOB in our study. In full-scale biological nutrient removal wastewater treatment plants, due to high loading rates, DO concentrations were observed to be 1–2 mg/L in aerobic basins, which limited the diffusion of oxygen in the activated sludge flocs with larger diameters. Therefore, the aerobic region was limited to the surface layer, and an anoxic zone could exist inside the flocs (Takacs and Fleit 1995; De et al. 1998; Li and Bishop 2003). The anoxic zone created an environment in which the reduction of nitrate by NaRB could take place. Excess substrate was supplemented for NOB and the percentage of NOB in the nitrifying community in the flocs increased.

As a conclusion, since the sequential oxidation of ammonium to nitrite then to nitrate, the growth balance between AOB and NOB plays a key role in optimization of nitrifying community. If the percentage of AOB is higher than that of NOB, and the ammonia-oxidizing rate is higher than nitrite-oxidizing rate, nitrite as an intermediate will be accumulated. Nitrite in the effluent will consume the oxygen in the receiving water body, making it toxic to aquatic organisms. Furthermore, high nitrite is positively correlated with N_2_O emissions from aerobic zones of activated sludge in the presence of low DO concentration (Ahn et al. 2010). N_2_O is a greenhouse gas and has a significant effect on globe warming. However, fortunately, in the WWTPs surveyed, NOB numbers were 1.72–5.87 times as abundant as AOB, and NURs were 1.1–2.0 times higher than AURs. A large nitrite oxidation pool could be established in a nitrifying community. High nitrite oxidation potential guaranteed nitrite, produced from ammonium oxidizing by AOB, will be quickly used by NOB. No intermediate was accumulated. Finally, a conceptual model was constructed to describe the bacterial growth balance in a nitrifying community which can explain the higher percentage of NOB in WWTPs. However, it should be investigated further in the future work.

## Additional file

**Additional file 1.** Additional figures.

## Abbreviations

BNR: biological nutrition removal
WWTPs: wastewater treatment plants
FISH: fluorescence in situ hybridization
AOB: ammonia-oxidizing bacteria
NOB: nitrite-oxidizing bacteria
GHG: greenhouse gas
OUR: oxygen uptake rates
SUR: substrate uptake rates
AUR: ammonium uptake rate
NUR: nitrite uptake rate
MLSS: mixed liquor suspended solids
MLVSS: mixed liquor volatile suspended solid
DO: dissolved oxygen
CLSM: confocal laser scanning microscope
TN: total nitrogen
SND: simultaneous nitrification and denitrification
SRT: sludge retention time
HRT: hydraulic retention time
