# Supplementary material for: Nitrite oxidizing bacteria (NOB) dominating in nitrifying community in full-scale biological nutrient removal wastewater treatment plants
Source: AMB Express. 2017 Jan 23;7:25. doi: 10.1186/s13568-017-0328-y (PMC5256632; doi:10.1186/s13568-017-0328-y)
Supplement: Supplementary file 1 — Additional file 1. Additional figures. [file 13568_2017_328_MOESM1_ESM.pdf]

Supplementary Figures for

Nitrite oxidizing bacteria (NOB) dominating in nitrifying community in full-scale biological nutrient removal wastewater treatment plants

AMB Express

Qian Yao[1, 2,] Dang-Cong Peng[1, 2]

[1]School of Environmental and Municipal Engineering, Xi'an University of Architecture and Technology, Xi'an, 710055, China;

[2]Key Laboratory of Northwest Water Resource Environment and Ecology, Ministry of Education, Xi'an, 710055, China.

Correspondence: Qian Yao, School of Environmental and Municipal Engineering, Xi'an University of Architecture and Technology, Xi'an, 710055, China

E-mail: qianyao@hotmail.com

Dengjiacun: W1

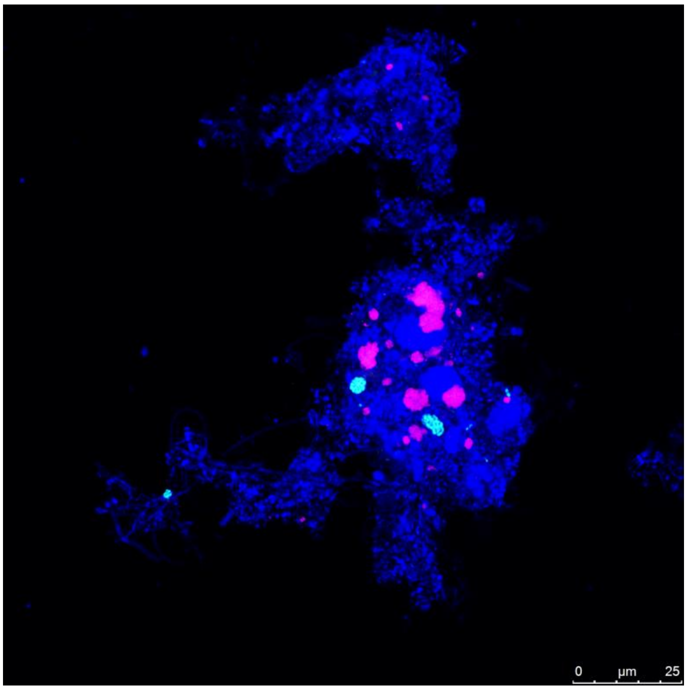

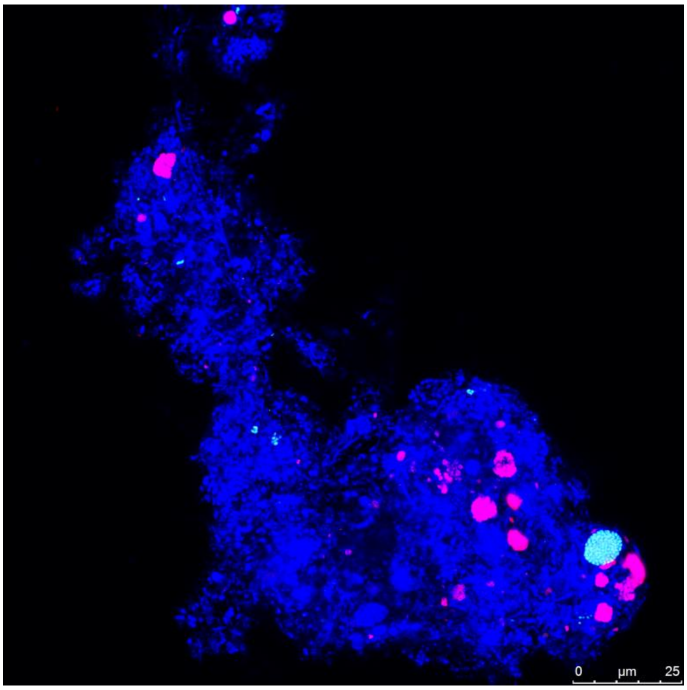

Dengcun: W2

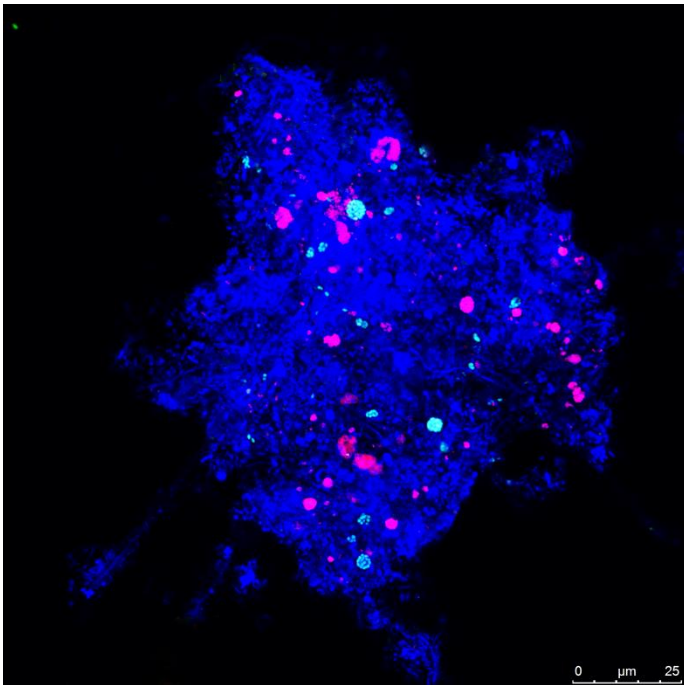

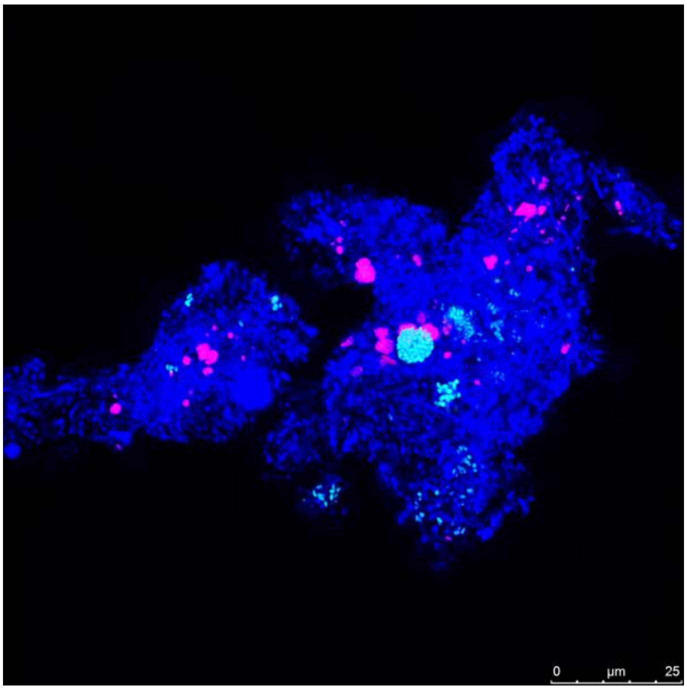

Beishiqiao: W3

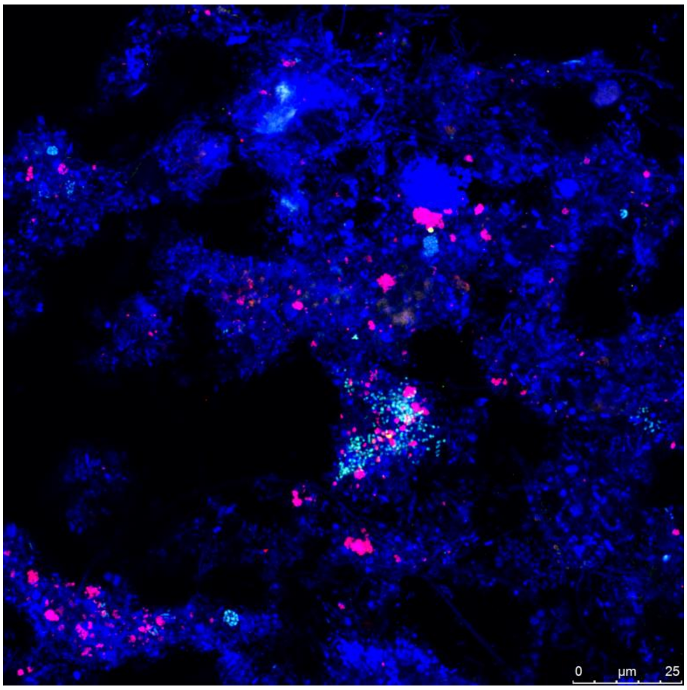

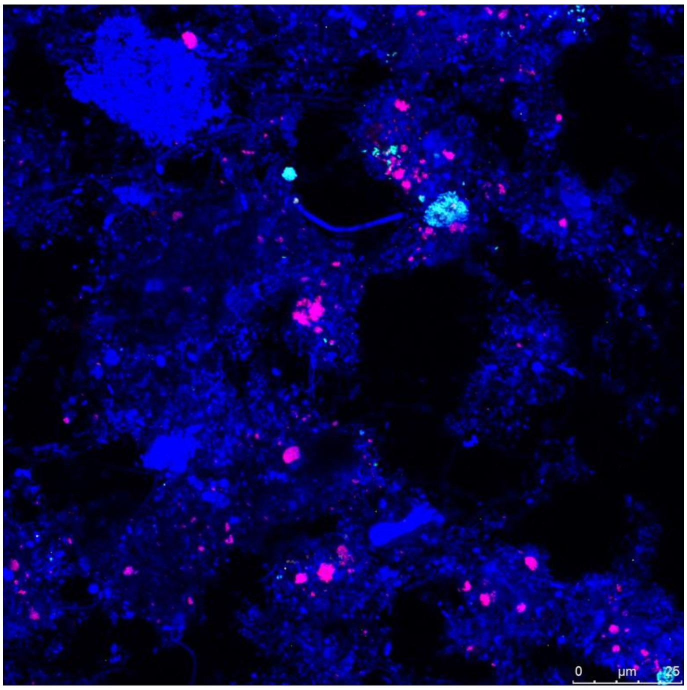

Bshiq: W4

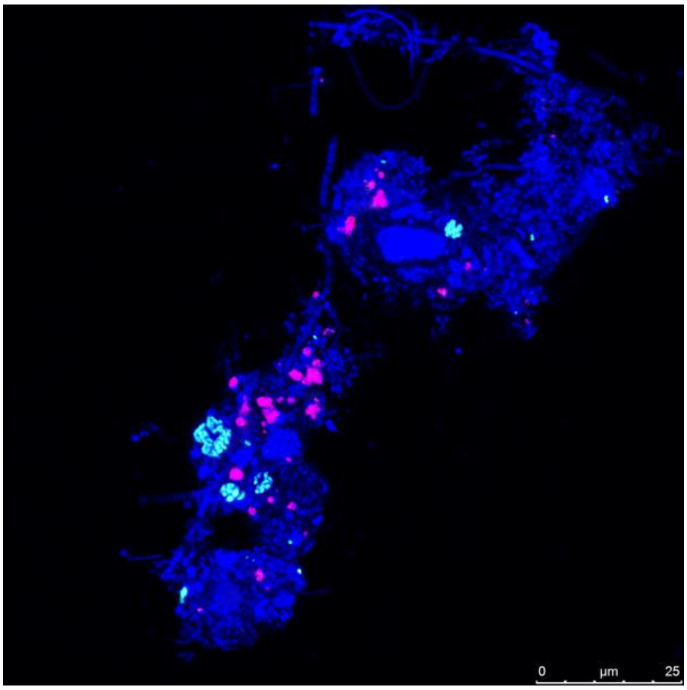

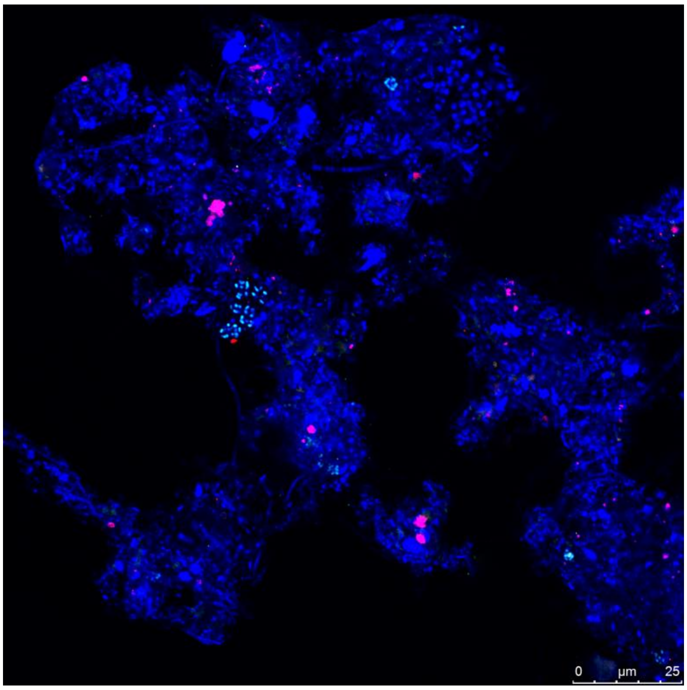

Fangzhicheng: W5

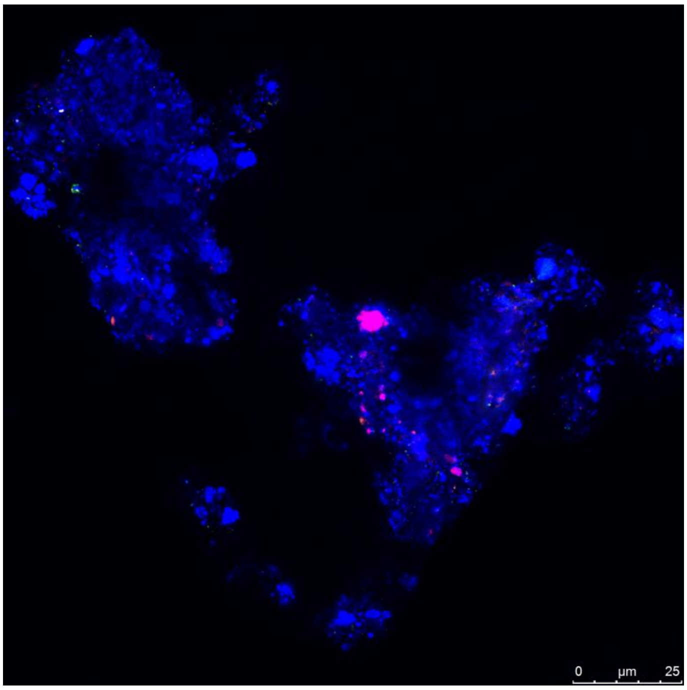

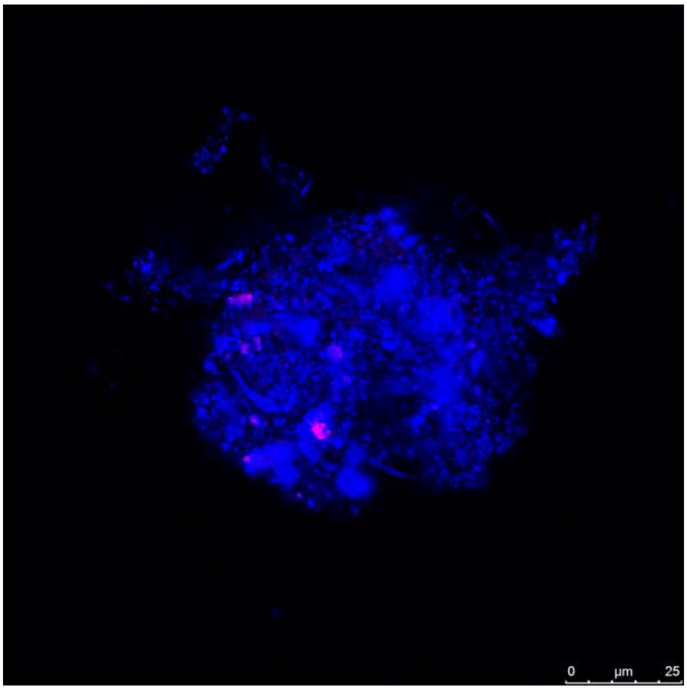

Dianzicun: W6

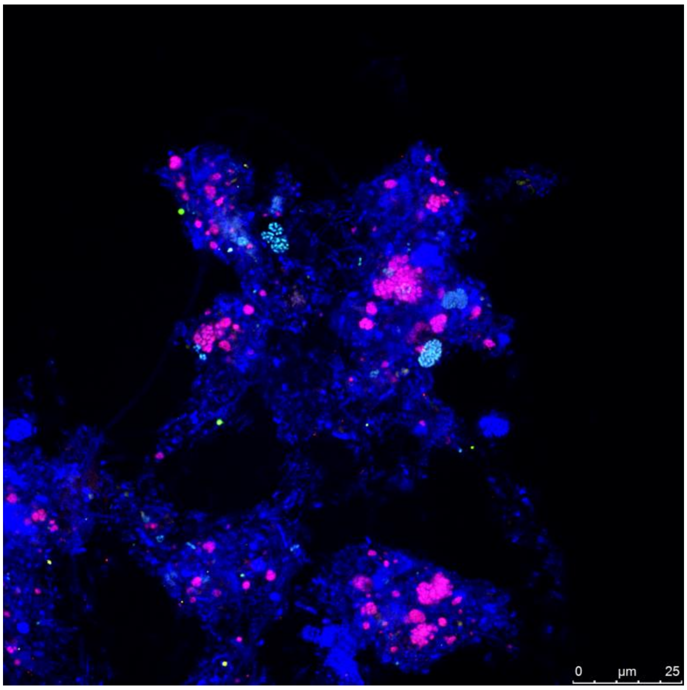

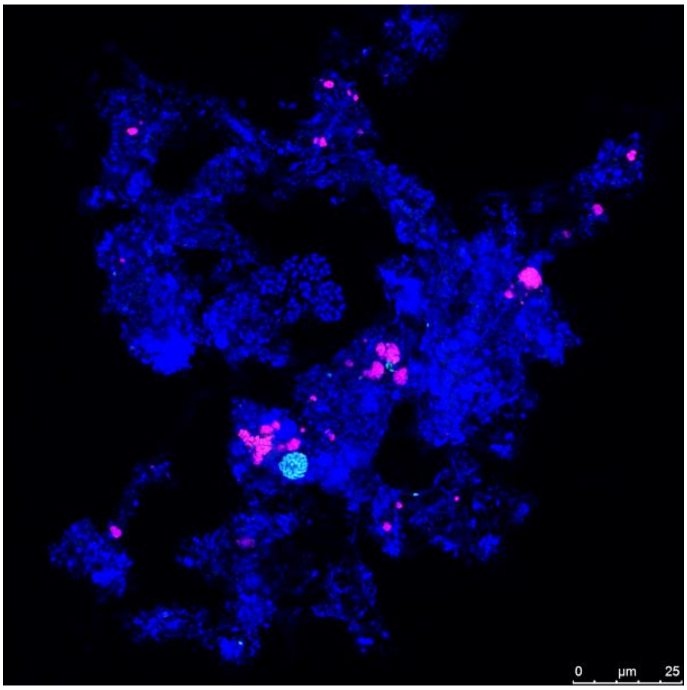

Yuanlecun: W7

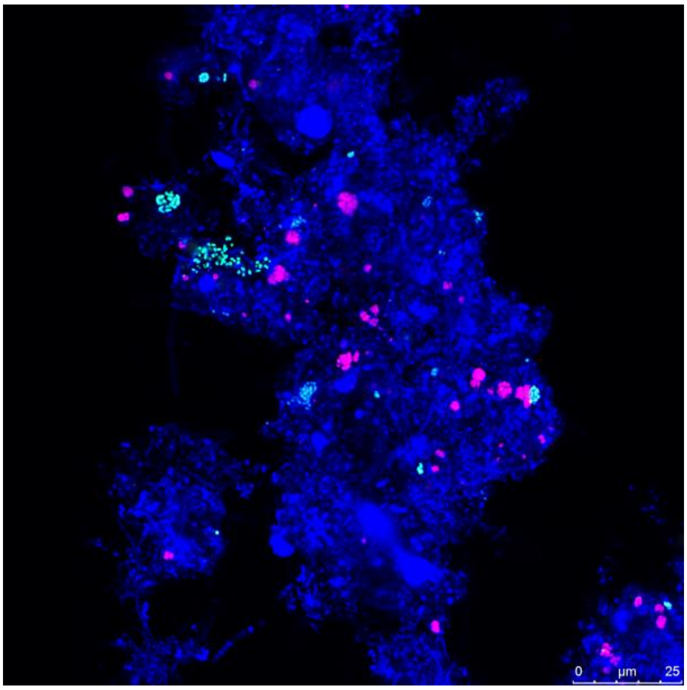

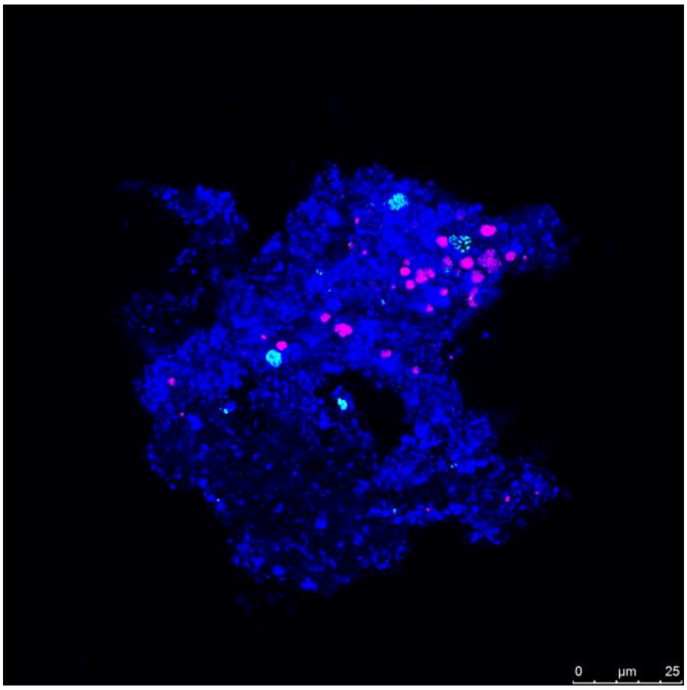

Liucunbao: W8

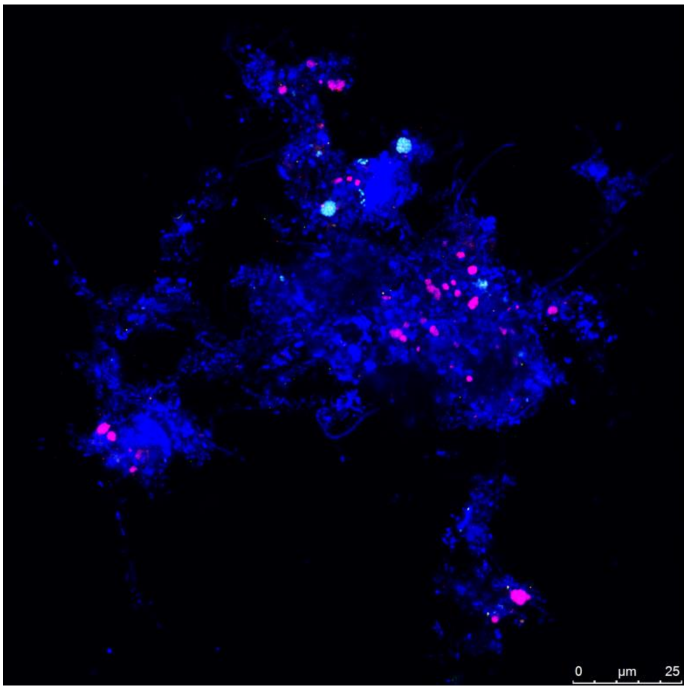

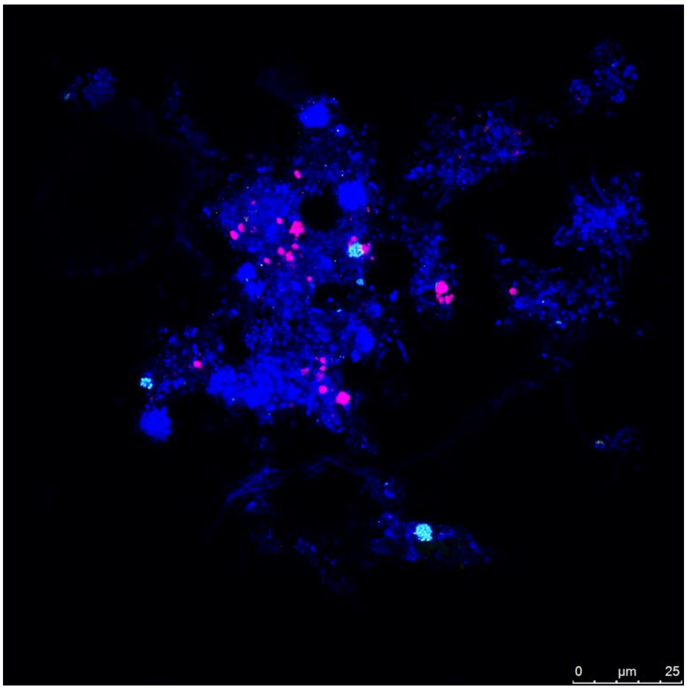

Gaoxin: W9

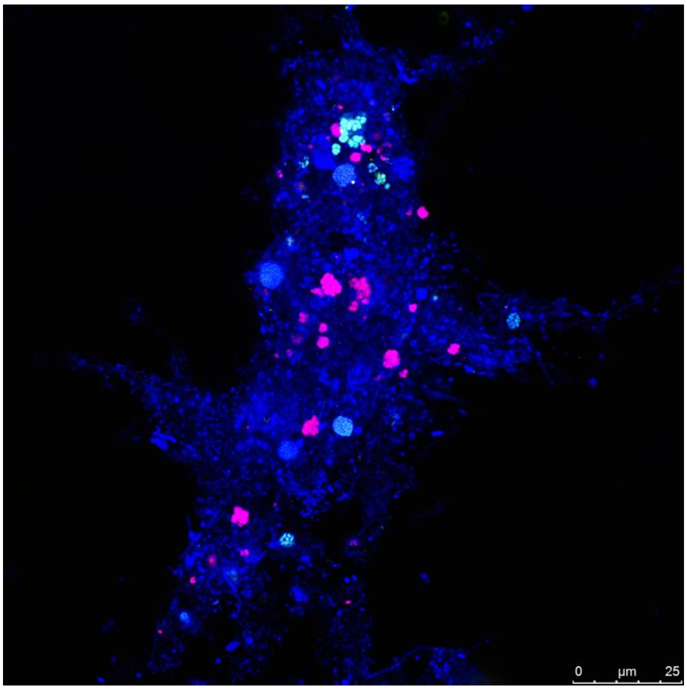

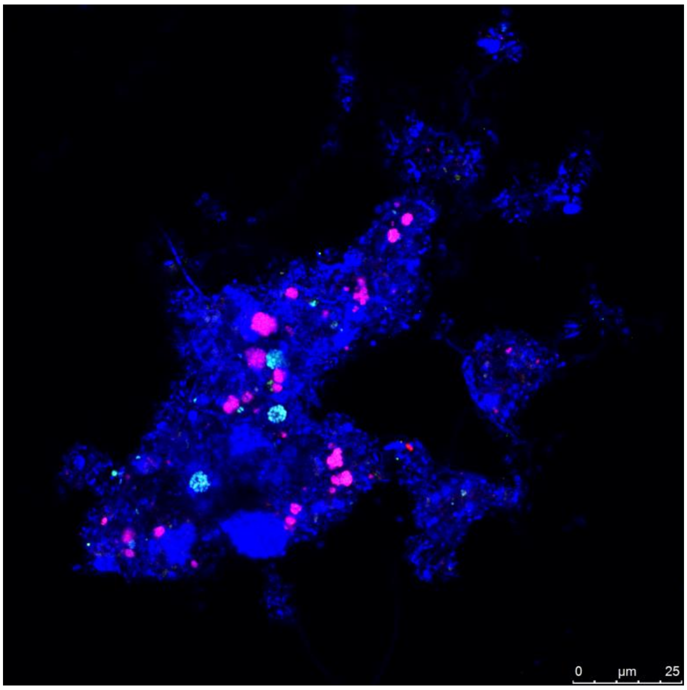

Changan: W10

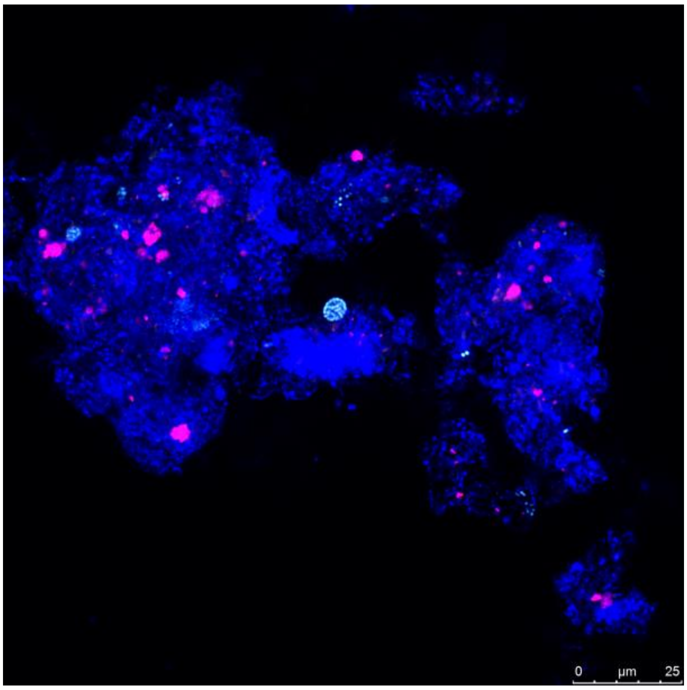

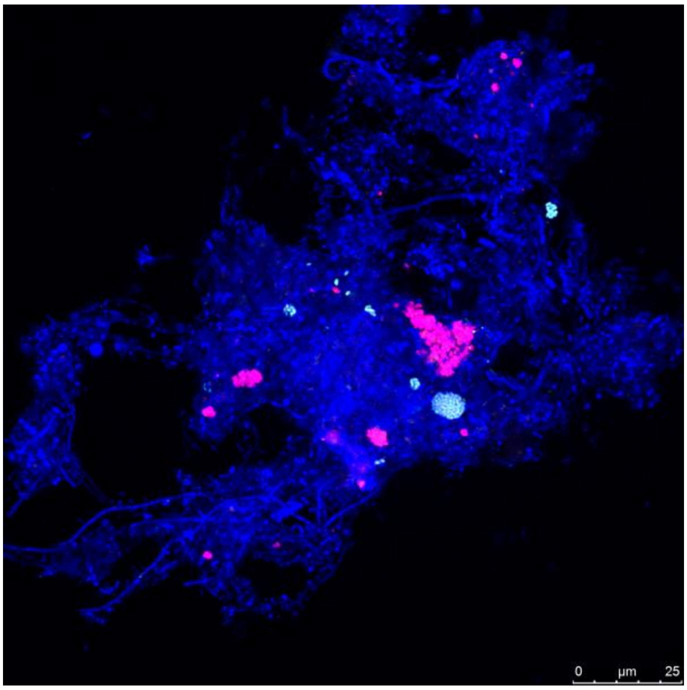

Figure S1. Community structure of nitrifying bacteria in 10 WWTPs In situ hybridization of activated sludge with Cy5-labeled probe EUBmix, Flous-labeled probe AOBmix and Cy3-labeled probe NOBmix. Blue: EUBmix-stained Eubacteria; cyan: AOBmix-stained AOB; carmine: NOBmix-stained NOB; Bar=25μm

Dengjiacun: W1

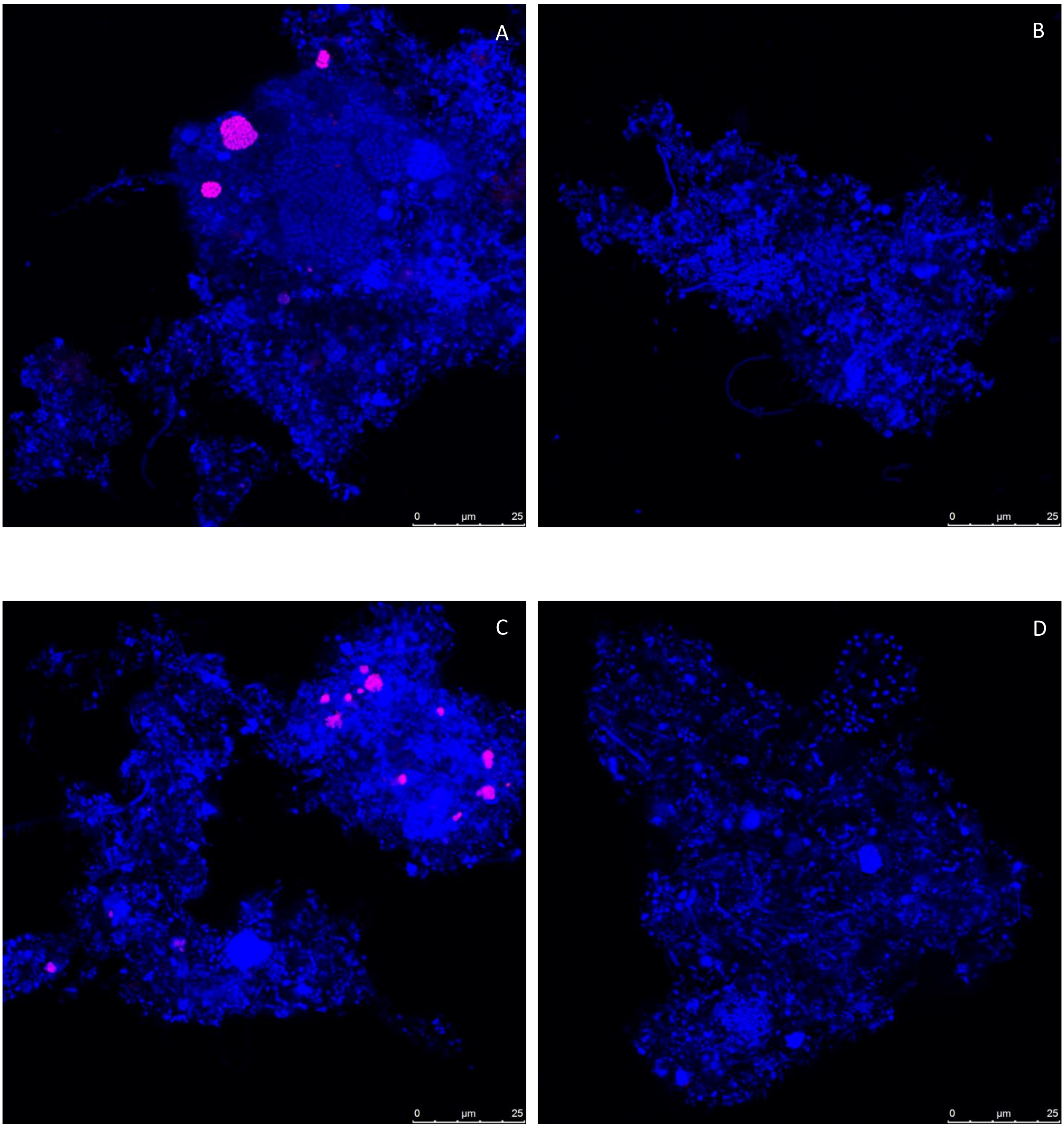

Dengcun: W2

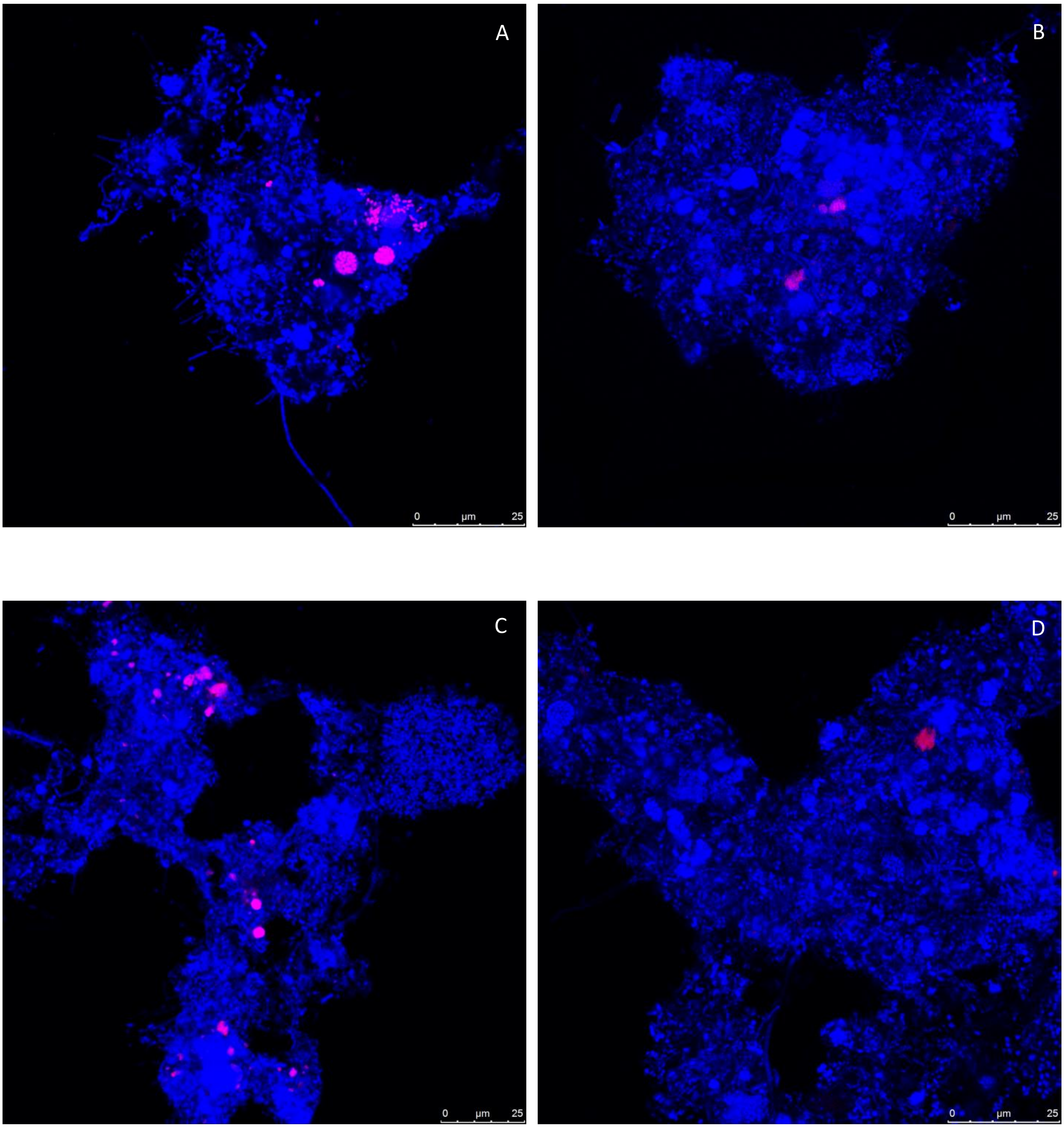

Beishiqiao: W3

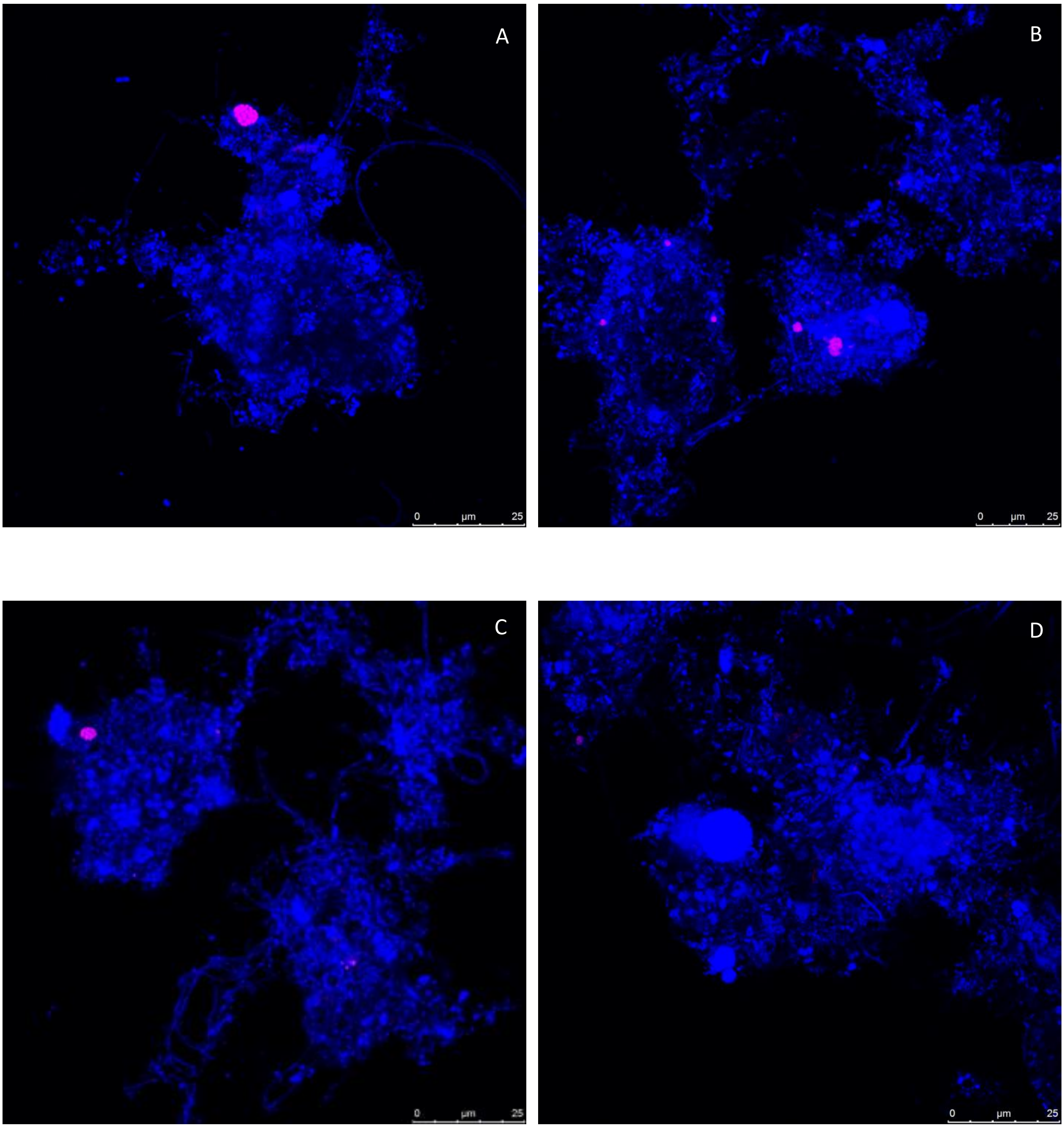

Bshiq: W4

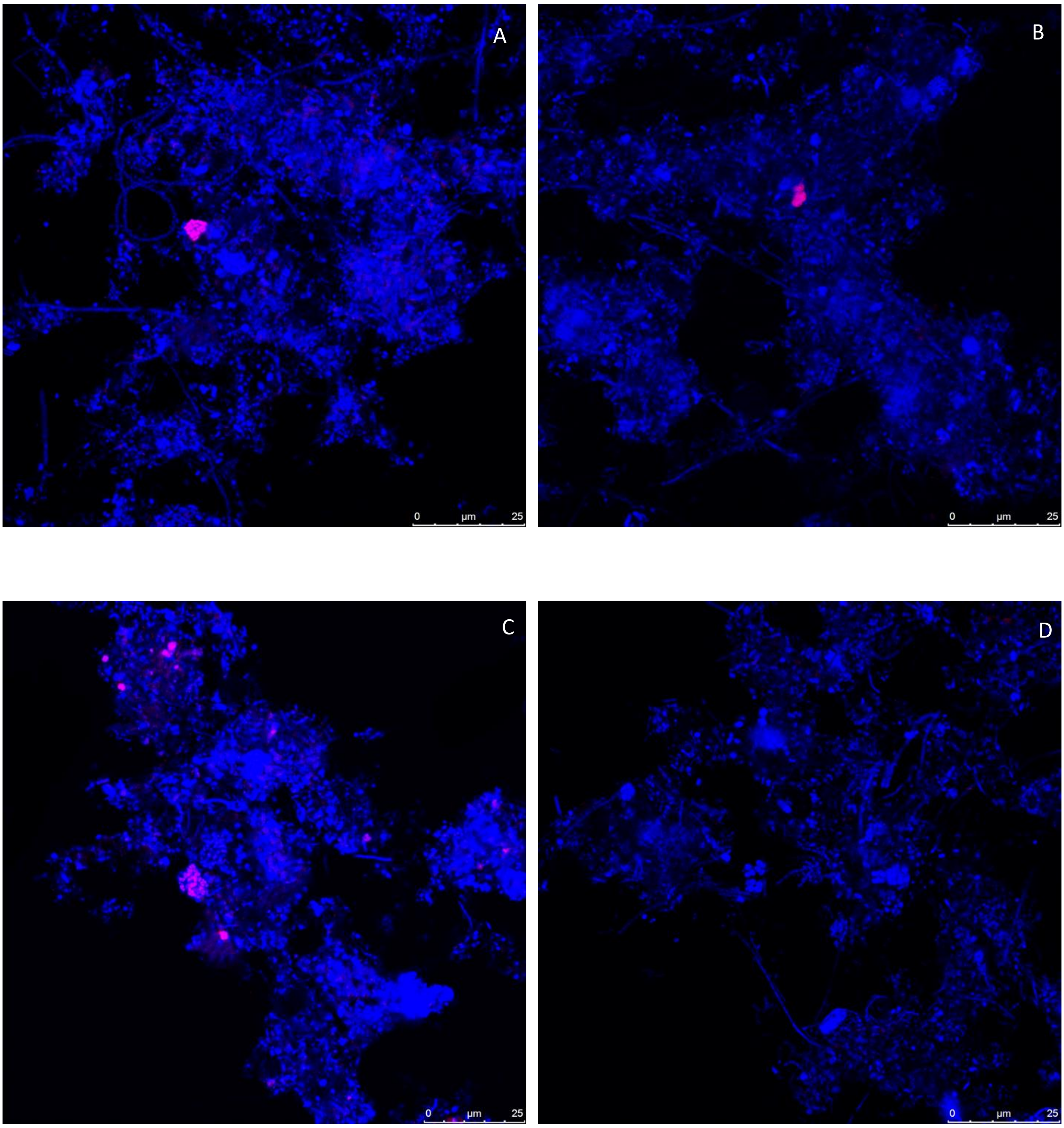

Fangzhicheng: W5

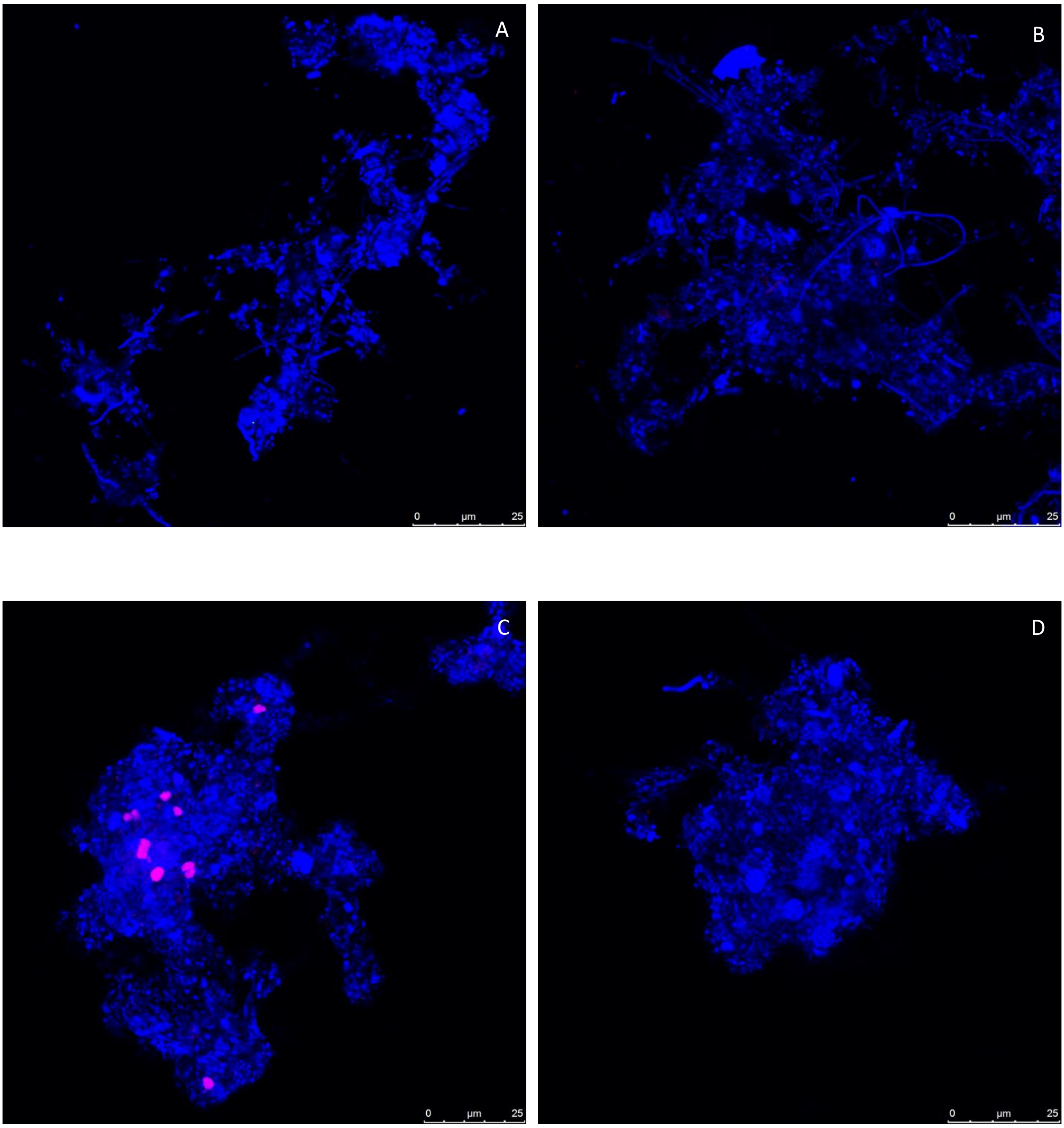

Dianzicun: W6

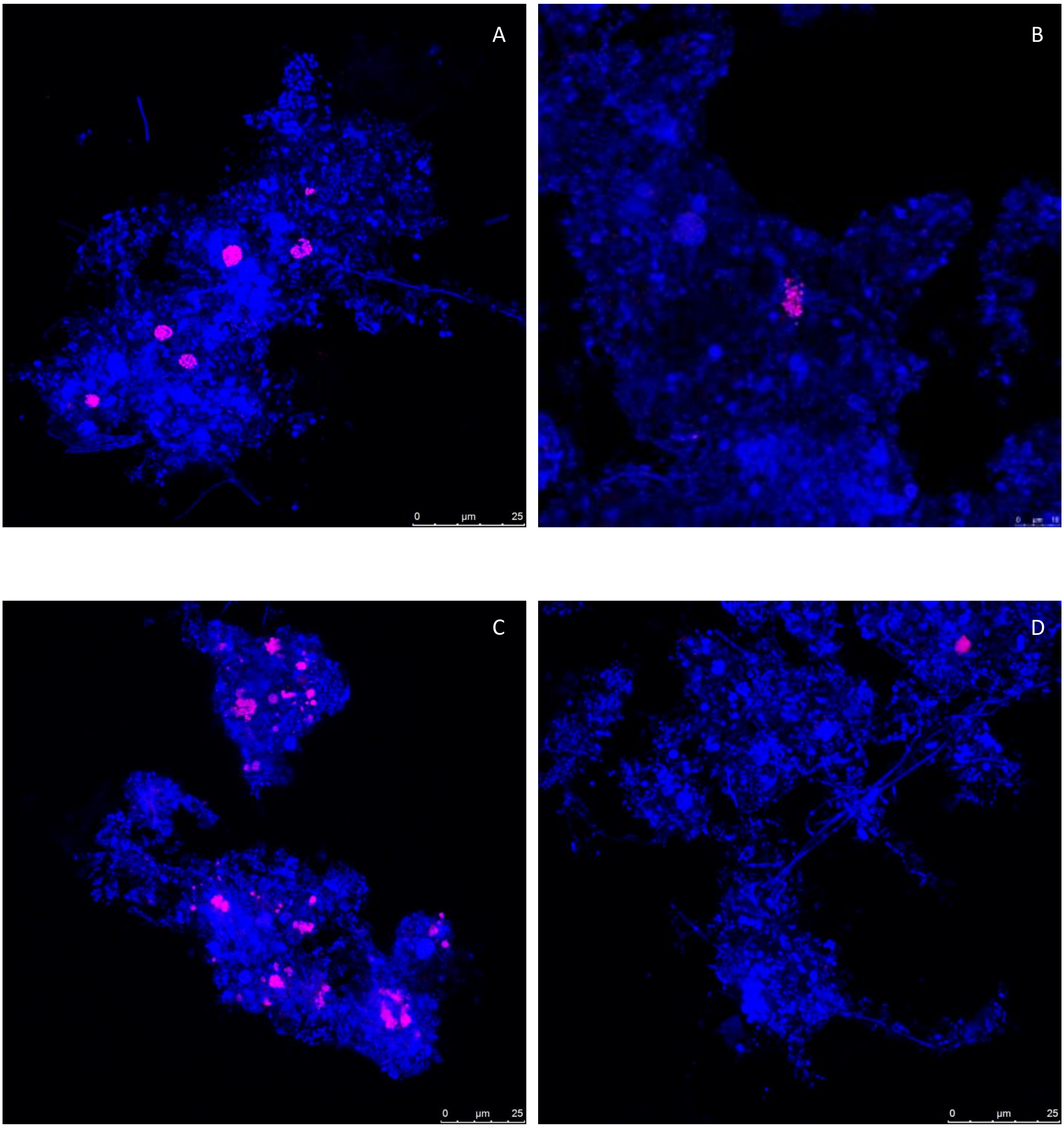

Yuanlecun: W7

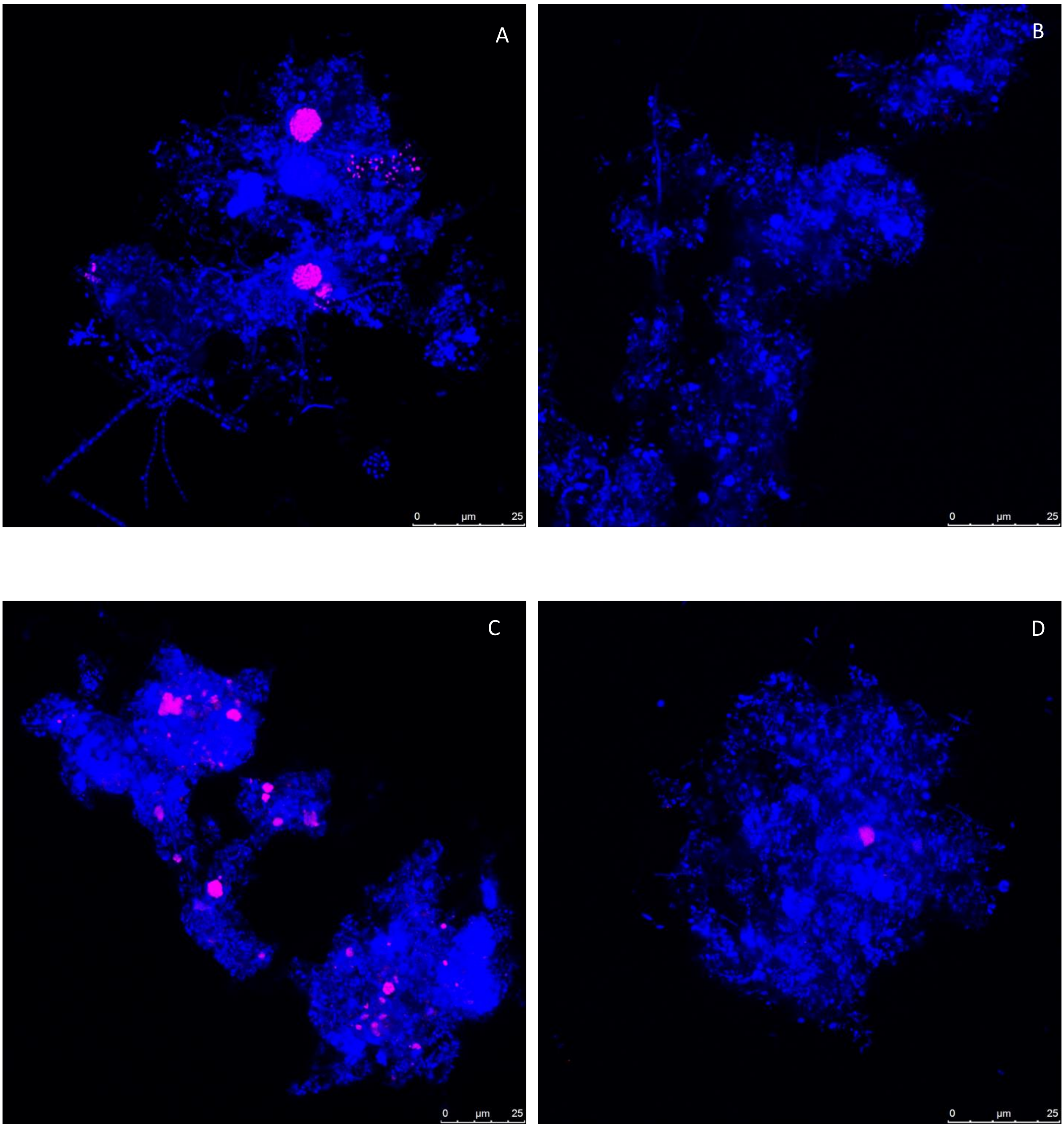

Liucunbao: W8

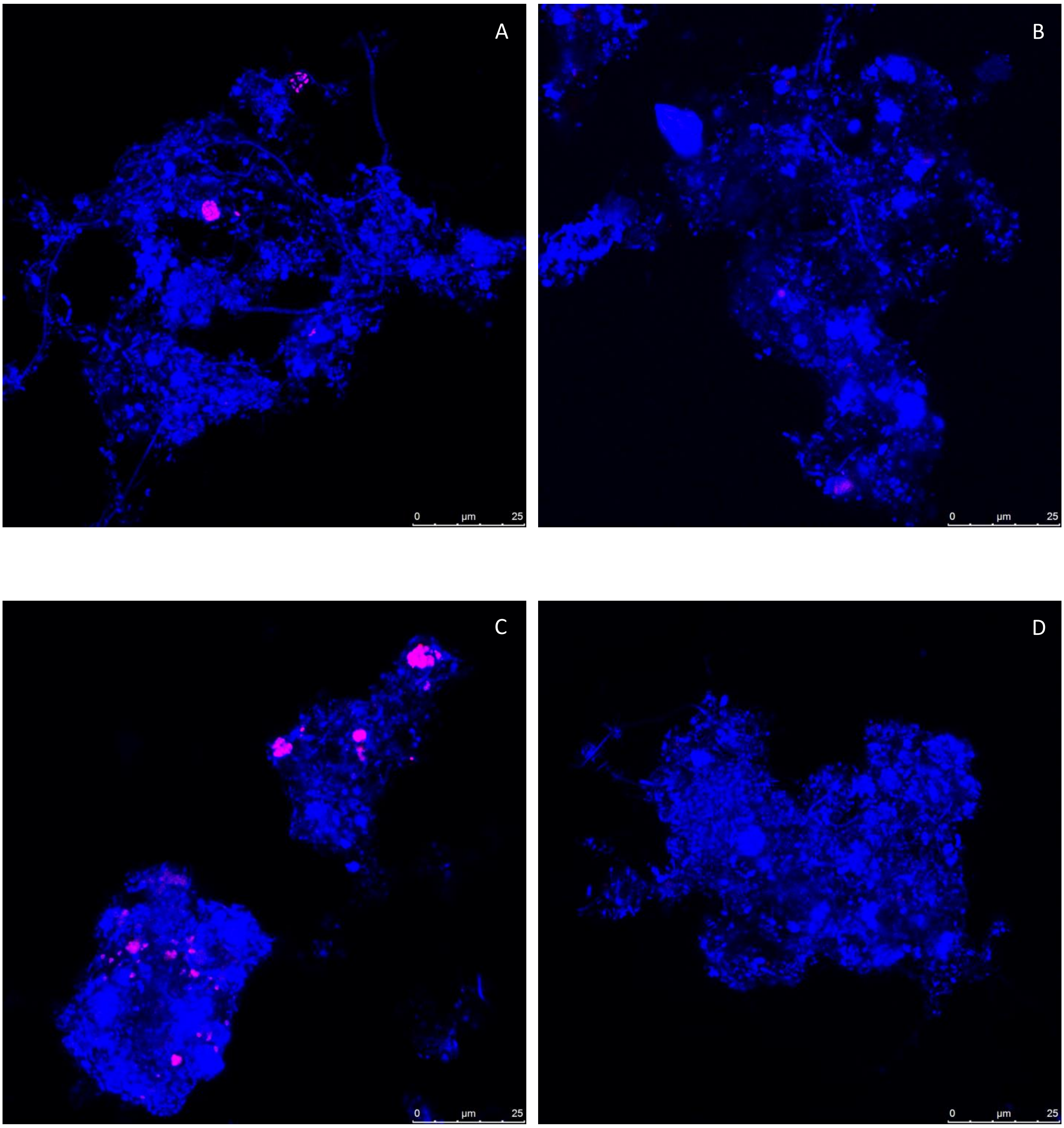

Gaoxin: W9

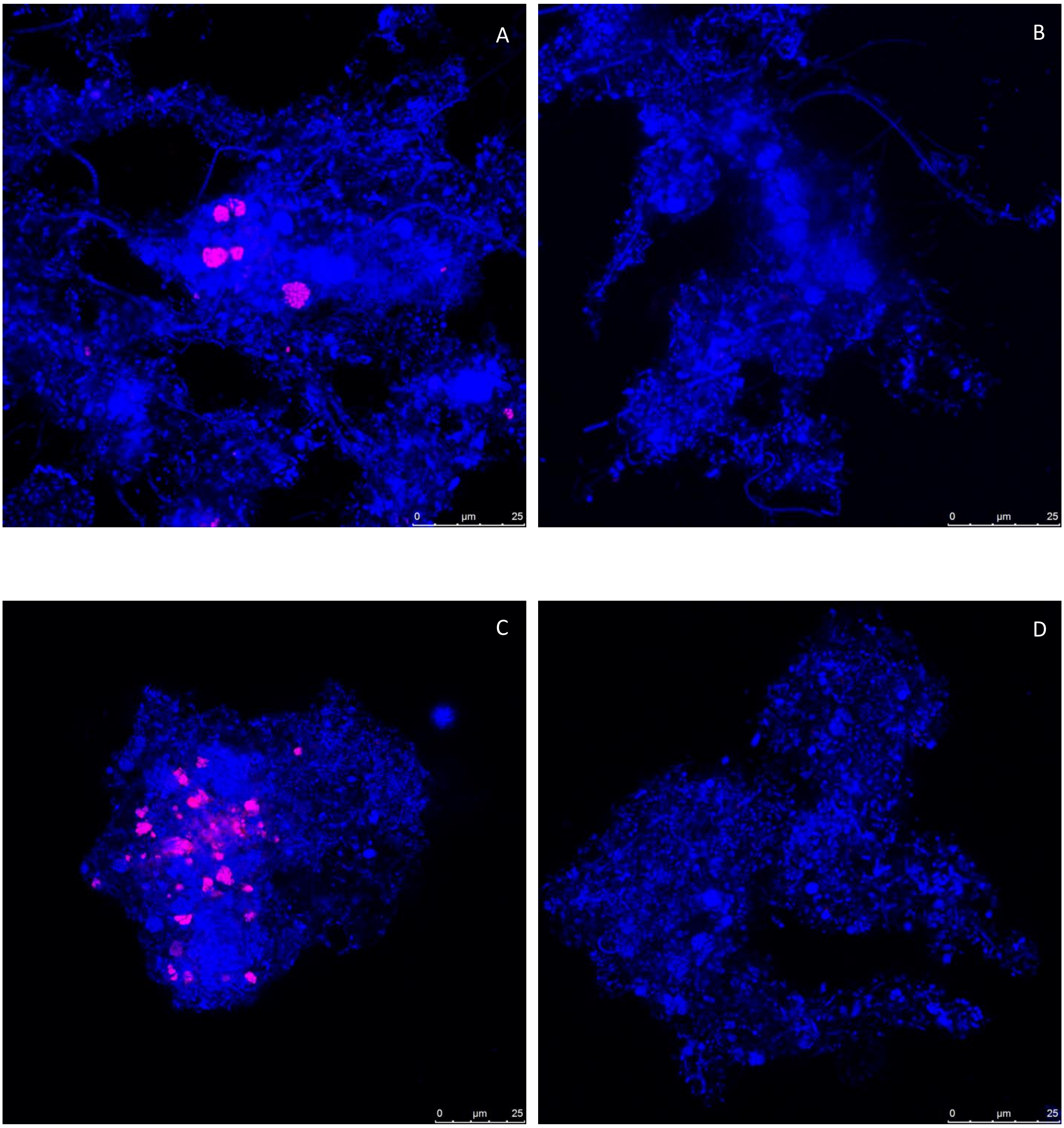

Changan: W10

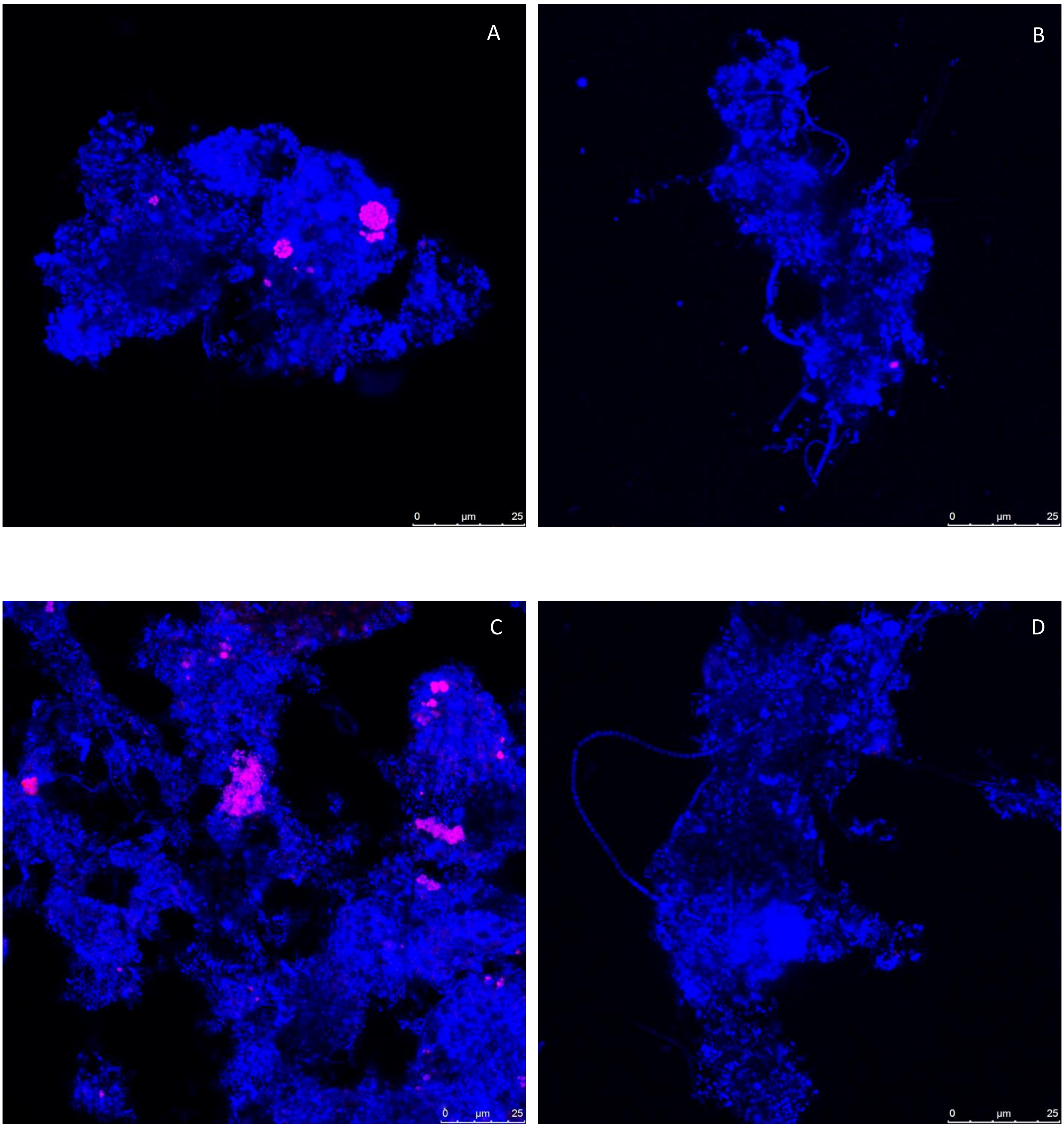

Figure S2. Four groups of nitrifying bacteria in activated sludge in 10 WWTPs. In suit hybridizations of actived sludge with Cy5-labeled probe EUBmix (Eubacteria), Cy3-labeled probe Nsm156 (Nitrosomonas)、Nsv443 (Nitrosospira)、Ntspa662 (Nitrospira) and Nit3 (Nitrobacter). Blue: Eubacteria; carmine: Nitrosomonas (A)、Nitrosospira (B)、Nitrospira (C) and Nitrobacter (D). Bar=25 μ m.

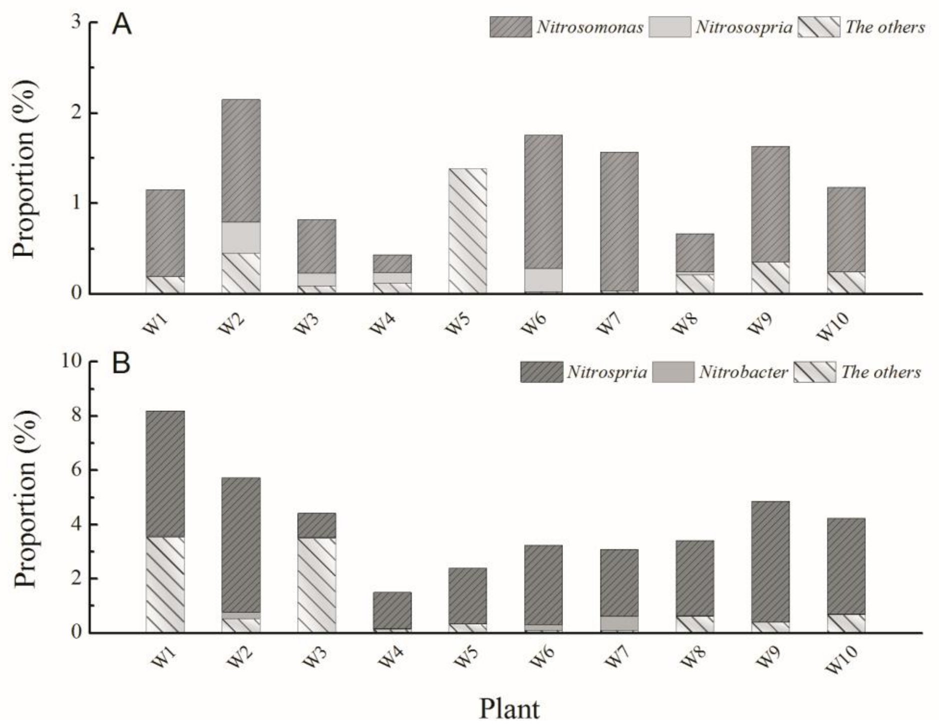

Figure S3. Community structure of nitrifying bacterial in 10 WWTPs (A: AOB; B: NOB).
